# Optimizing Molecular Descriptors for Reliable Adsorption Energy Prediction on Transition Metal Nanoclusters

**DOI:** 10.1021/acsomega.5c09138

**Published:** 2026-01-06

**Authors:** Lucas B. Pena, Felipe V. Calderan, Priscilla Felício-Sousa, Karla F. Andriani, Marcos G. Quiles, Juarez L. F. Da Silva, Breno R. L. Galvão

**Affiliations:** † 74355 Centro Federal de Educação Tecnológica de Minas Gerais, 30421-169 Belo Horizonte, MG, Brazil; ‡ Institute of Science and Technology, 28105 Federal University of São Paulo, 01109-010 São Paulo, SP, Brazil; § São Carlos Institute of Chemistry, 153988 University of São Paulo, Av. Trabalhador São-Carlense 400, 13560-970 São Carlos, SP, Brazil; ∥ Departament of Exact Sciences, State University of Santa Cruz, 45662-900 Ilhéus, BA, Brazil

## Abstract

Efficient catalytic processes are crucial for converting pollutant molecules into valuable products. Transition-metal nanoclusters show promise as a result of their tunable properties, but identifying active catalysts requires costly studies of the adsorption energetics. Machine learning offers a faster alternative, predicting adsorption energies when trained on suitable descriptors and relatively large density functional theory (DFT) data sets. This study evaluates the predictive power and transferability of two structural descriptors, the Coulomb matrix and the many-body tensor representation, on a diverse nanocluster-adsorbate data set using the random forest regression algorithm. Both descriptors achieved a mean absolute error of 0.05 eV in test data, but performance dropped significantly on an external generated set with unprecedented examples. Adding a simple electronic feature, the number of unpaired electrons of the adsorbate improved generalizability, even though with higher mean absolute errors compared to the original data set, highlighting the dependence on the training data.

## Graphical abstract

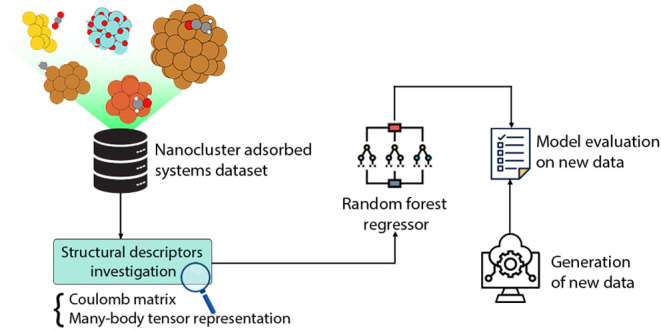

## 1 Introduction

Catalytic processes represent one of the most promising strategies for developing industrially and energetically viable chemical routes to convert pollutant molecules into value-added chemicals.^1,2^ Particularly, transition metal nanoclusters have emerged as highly effective catalysts for reactions such as CO_2_ reduction,^3–5^ CH_4_ oxidation,^6–8^ and reactions of oxygen and hydrogen evolution reactions,^9–11^ primarily due to their unique and tunable catalytic properties. By controlling structural and electronic characteristics, nanoclusters can be tailored for specific catalytic pathways, thus improving activity and selectivity toward desired products.^12^ However, the rational design of nanocluster catalysts requires a fundamental understanding of the adsorption behavior of reactant species on their surfaces, as adsorption is the first and most crucial step in the catalytic reaction pathway.

The adsorption is the initial step that modulates the activation of the reactant molecules, as well as the entire reaction mechanism, by influencing subsequent steps: bond dissociation, intermediate formation, product desorption, and so on. Therefore, an atomistic understanding of the adsorption process is crucial to unraveling the full catalytic pathways, particularly in terms of energetics, and hence it is fundamental to guide the rational design of more efficient and selective catalysts.^13^ However, experimentally determining adsorption energies is often time-consuming and resource intensive, as it requires sophisticated synthesis procedures, surface characterization, and adsorption measurements.^14^ Consequently, first-principles approaches based on density functional theory (DFT) have become widely used to investigate adsorption phenomena on catalytic surfaces with high accuracy. These approaches not only provide reliable information on the adsorption mechanism, but also enable the screening of a large number of nanocluster configurations, particularly when coupled with advanced sampling strategies such as genetic algorithms^15–17^ or clustering techniques.^18–21^

However, the complex nature of adsorption, arising from the numerous possible sites and configurations for each adsorbate, makes comprehensive sampling computationally demanding, requiring a careful balance between accuracy and computational efficiency. To overcome this bottleneck, machine learning (ML) methods have recently emerged as a promising alternative to predict adsorption energies with significantly lower computational cost.^22^ These models can be trained on a relatively small set of DFT-calculated adsorption energies and subsequently employed to predict properties for a much larger set of configurations. A critical aspect in developing such models is feature engineering, that is, the process of selecting and constructing descriptors that effectively encode the essential physical and chemical information on a system into a ML-friendly format.^23^ Descriptors are often grouped into structural features, which capture geometric information from Cartesian coordinates,^24–28^ atomic or elemental features, which are based on periodic trends,^29–32^ and electronic features, which may require less expensive or fewer DFT calculations for their determination.^33–35^

Recent advances in ML for nanocluster catalysis have demonstrated that structural descriptors can effectively encode adsorption systems, enabling accurate predictions of adsorption energies.^36–38^ However, these advances are often based on data sets with important limitations. In many cases, data sets are restricted to specific systems, for instance, a single or a few nanocluster compositions,^24,39,40^ nanoclusters with highly constrained geometries,^41,42^ or a small set of interacting adsorbate species.^25,32,33,43^ As a result, the generality and transferability of models trained on these data sets remain uncertain when applied to different catalytic systems or adsorption environments.

A critical challenge in this field is the high computational cost associated with the generation of large, diverse, and high-quality data sets using DFT calculations. Performing DFT calculations specifically tailored for ML model training can quickly become prohibitive, particularly when the need to cover different compositions, adsorbates, and structural motifs. An alternative approach, which motivated this work, is to leverage existing DFT calculations originally performed for traditional catalysis or adsorption studies, which were not explicitly designed for ML purposes. Although such data sets often lack systematic sampling or uniform coverage of chemical space, they represent a valuable and underutilized resource for building predictive models with practical applicability.

In this context, our work aims to investigate whether a reliable ML model for adsorption energy prediction can be constructed from heterogeneous, high-cost DFT data originally generated for physical and chemical insight, rather than for ML training. We also explore the inherent limitations of structural descriptors under these conditions, particularly their ability to generalize beyond the types of structures and adsorbates present in the training data. To address this, we assembled a diverse data set comprising different nanocluster compositions, sizes, and geometries interacting with a variety of adsorbates. Two state-of-the-art structural descriptors, the Coulomb matrix (CM)^44^ and the many-body tensor representation (MBTR),^45^ were systematically evaluated in conjunction with the random forest regressor (RFR) ML algorithm.^46^ The RFR with both descriptors achieve prediction errors comparable to the intrinsic uncertainties of DFT,^47–49^ but their generalizability is critically dependent on sample representativeness in the training set.

To further investigate these limitations, we applied the trained RFR with the CM and LMBTR descriptors to a newly generated external data set composed of the most representative nanocluster in the training data (Cu_13_) combined with five different adsorbates. The results reveal that, while electronic features, such as the number of unpaired electrons, improve the predictions across different adsorbates, the models still struggle with configurations that are structurally distinct from the training data. This highlights that although leveraging existing DFT data is a promising pathway, especially when generating new data sets is computationally prohibitive, ensuring adequate structural diversity remains essential for robust and transferable ML models in catalysis.

## 2 Theoretical Approach and Computational Details

Figure 1 provides a schematic representation of the data processing and model development pipeline used in this study. The process begins with the construction and characterization of the data set,^50–57^ as detailed in Section 2.1. The data were separated into two distinct subsets: one for the optimization of structural descriptors as discussed in Section 2.2, and another for the hyperparameter tuning of the RFR, as elaborated in Section 2.3, using the optimal featurization protocols already established.^46,58^ For the final evaluation of the model, an external test set was designed and automatically generated, as explained in Section 2.4, while DFT calculations were executed according to Section 2.5 to acquire the target values and allow comparison with model predictions. Moreover, an electronic characteristic, specifically the number of unpaired electrons in the adsorbate, was integrated into the CM eigenspectrum as an additional descriptor. This integrated approach ensures rigorous model training, precise performance evaluation, and reliable generalization assessment through the application of the external test set.

**Figure 1:**
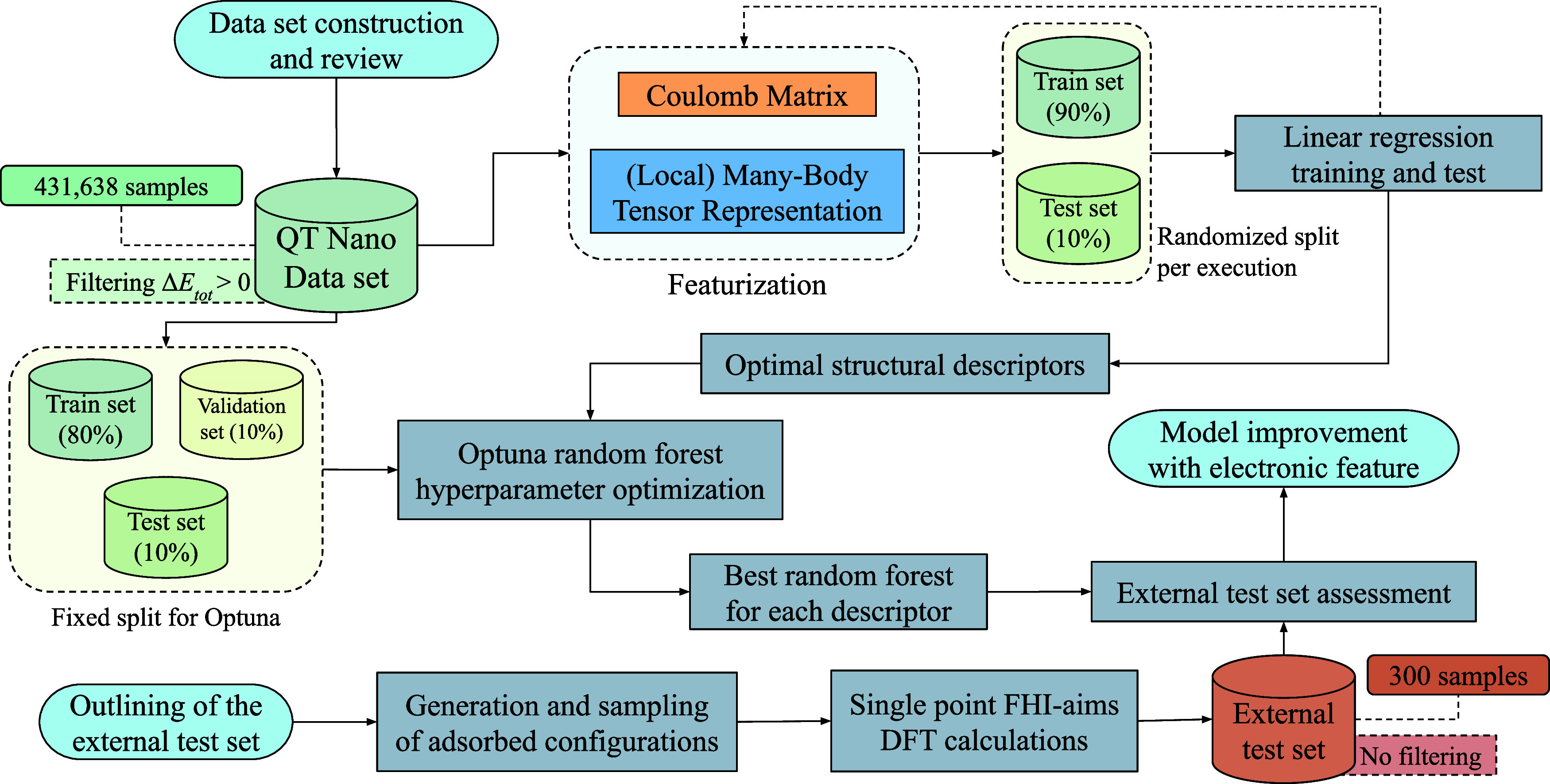
Schematic representation of the data processing and model development pipeline that includes the construction of data sets, the optimization of descriptors and models, and the subsequent evaluation using an external test set.

### 2.1 Quantum Chemistry Data Set

The data set used in this research was collected from previous and ongoing studies conducted by the QTNano group,^59^ specifically focusing on selected adsorbed species in metal and oxide nanoclusters.^50–57^ These investigations encompass a wide range of adsorbate nanocluster systems, including both metallic and oxide-based nanoclusters, characterized by variations in size, geometry, and chemical composition. Furthermore, the data set comprises a broad selection of adsorbate species pertinent to catalytic reactions, particularly concerning the CH_4_ and CO_2_ conversion pathways. However, the DFT calculations used in this work were conducted purely for investigating adsorption phenomena and were not originally intended for ML model development. By employing these data in the development of the ML model, we aim to investigate the feasibility and limitations of repurposing existing high-cost computational data for predictive modeling, assessing how well such data sets can support generalizable and reliable machine learning algorithms without requiring extensive new calculations.

Figure 2 provides an overarching representation of the sizes, compositions and adsorbate species of nanoclusters incorporated into our data set. As shown, the collection spans 15 different compositions and 14 nanocluster sizes, paired with 43 unique adsorbate species. Although adsorbates are not uniformly distributed across all cluster types, this chemical diversity markedly exceeds the scope of most previous studies, many of which are restricted to a single cluster size, a narrow range of compositions or geometries, or even a single adsorbate.^24,25,39^ A detailed breakdown of all adsorbed systems and their literature sources can be found in the Supporting Information (SI).

**Figure 2:**
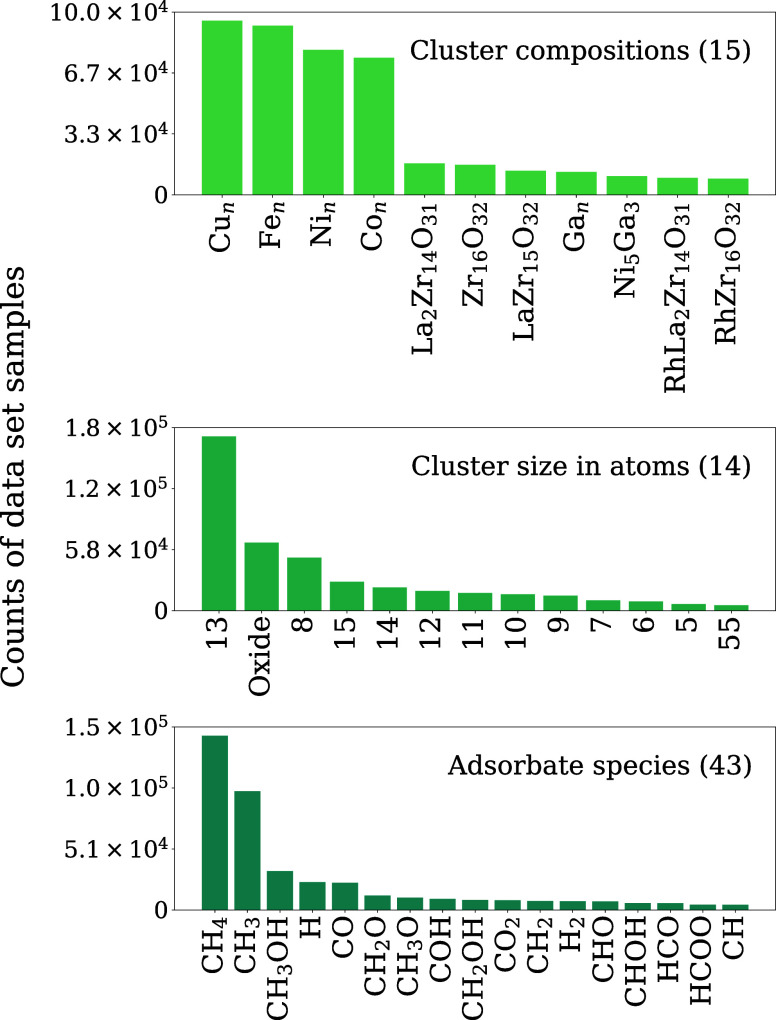
Data set breakdown by nanocluster size, composition, and adsorbate species. Categories that include less than 1% are omitted for clarity. The total number of categories present in the data set are provided in parentheses for each graph.

The entries in this data set correspond to Cartesian coordinates of the adsorbed systems (.*xyz* file), together with their relative total energy (Δ*E*_tot_), which is defined as

1 $$\Delta E_{\mathrm{tot}}=E_{\mathrm{tot}}^{\mathrm{system}}-\left(E_{\mathrm{tot}}^{\mathrm{nanocluster}}+E_{\mathrm{tot}}^{\mathrm{adsorbate}}\right)$$

where *E*_tot_^system^ is the total energy of the adsorbed system, and *E*_tot_^nanocluster^ and *E*_tot_^adsorbate^ are the total energies of the reference nanocluster and the adsorbate in the gas phase, respectively.

The data set comprises a total of 431,638 samples, each derived from DFT geometry optimizations performed using the FHI-aims package,^60,61^ with further elaboration provided in Section 2.5. By including all steps of the geometry relaxation process, the data set encompasses not only the final optimized structures but also less stable molecular configurations. To ensure that the model is concentrated solely on pertinent adsorption scenarios, samples with Δ*E*_tot_ > 0 were excluded from the data set, that is, a total of 19.897 samples or 4.6% of the initial data set were excluded.

### 2.2 Featurization

To ensure reproducibility and meaningful comparison between different adsorbed systems, it is essential to define how the structural data represented in Cartesian coordinates *xyz* can be transformed into model-ready feature vectors. Although *xyz* files are sufficient for quantum chemistry calculations, they are not ideal for ML applications, as the raw Cartesian coordinates are not inherently invariant to rotations, translations, or permutations of atom ordering. For instance, the same chemical system can be declared in multiple ways, as either the atom ordering or the spatial coordinates can vary due to arbitrary choices in labeling or the origin of the coordinate system. However, these variations should not affect the underlying physical or chemical properties that the machine learning model is meant to capture.^23^

In ML tasks, it is critical that each sample is represented by a fixed-length feature vector with a consistent ordering and meaning of features across the data set. Variations in vector size or representation introduce ambiguity and hinder model training, as most learning algorithms assume that each input vector corresponds to a point in the same high-dimensional space. Therefore, raw data *xyz* must be transformed into invariant size-consistent descriptors to ensure robust and transferable model performance.

#### 2.2.1 Model Evaluation

The mean absolute error (MAE) metric was used to evaluate the performance of the regression algorithms used and to guide the optimization of structural descriptors in modeling the adsorbed systems into consistent feature vectors. For any given set of examples, the MAE is calculated as

2 $$\mathrm{MAE}=\frac{1}{N}\sum_{i=1}^{N}\left|y_{i}-{ŷ}_{i}\right|$$

where *N* is the number of samples in the evaluated set, *ŷ*_*i*_ denotes the predicted value and *y*_*i*_ represents the corresponding real value for the sample *i*. The MAE quantifies how closely the predictions align with true values, and is expressed in the same unit as the property being evaluated (Δ*E*_tot_), allowing for direct comparison among different descriptor configurations.

For all experiments on modeling the data set to predict Δ*E*_tot_, the data set was split into training and testing sets to fit regression algorithms and evaluate performance using the MAE metric. In each trial, both sets were generated by randomly sampling without replacement from the filtered data set that only contained samples with Δ*E*_tot_ > 0. The training set comprised 90% of the data, while the test set contained the remaining 10%. This test set size was deemed sufficient to assess model generalizability, given the substantial diversity of nanocluster-adsorbed systems represented in the data set.

#### 2.2.2 Adsorbed System Modeling

The energy of interaction of molecular species with substrate atoms on surfaces or finite-size molecular systems depends mainly on a few atoms around the interaction point. This is due to the localized nature of the chemical bonding, which decays rapidly with distance and is typically dominated by first- and second-neighbor interactions. Motivated by this observation, all structural descriptors were constructed using only local structural information around the adsorption site, restricted by a user-defined site_size parameter (*s*) as illustrated in Figure 3. This parameter retains only the *s* closest nanocluster atoms to the adsorbate, removing the remaining nanocluster structure from the featurization procedure. This localized approach concentrates the modeling on the adsorption region, enhancing both transferability and computational efficiency by reducing the dimensionality of the problem through decreasing the number of Cartesian coordinates and pairwise terms included in the calculations. Specifically, by restricting the modeling on the site atoms and the interacting adsorbate species, the approach facilitates the efficient prediction of Δ*E*_tot_ for large systems, thus improving the scalability and broad applicability of the model in screening scenarios.

**Figure 3:**
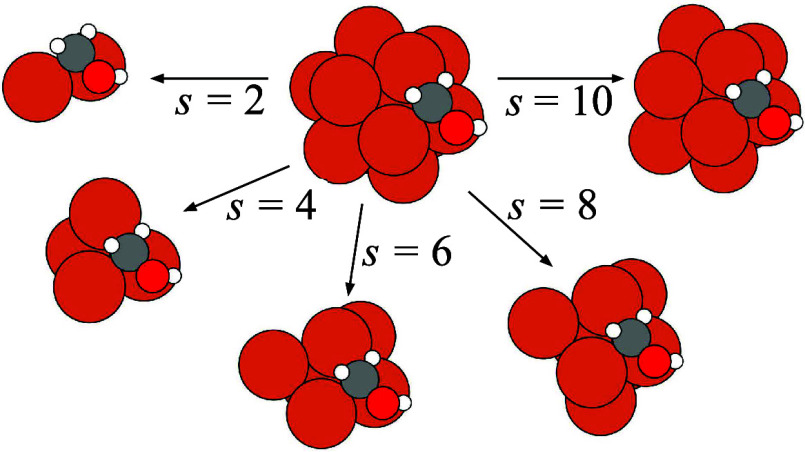
Constraining the Fe_13_/CH_2_OH adsorbed system structural information by selecting different numbers of site atoms (*s*) from the nanocluster.

With localized sample data, the structural descriptors transform Cartesian information into a model-ready feature vector according to defined user parameters, as described in the following Sections 2.2.3 and 2.2.4. The CM and MBTR are two levels of structural descriptors which differ in complexity and use of approximations for achieving a uniquely defined feature vector. To understand the influence of the parameter *s* combined with the functionalization processes of both CM and MBTR, linear regression algorithms were used. These algorithms were selected as baseline ML regressors because of their simplicity, ensuring that any improvements in predictive capability are attributed to the efficiency of the descriptors in representing the adsorption systems, rather than to the complexity of the regression algorithm itself. Therefore, enhancements can be attributed to the identification of optimal descriptor parameters within the defined search space for the selected structural descriptors.

#### 2.2.3 Coulomb Matrix Eigenspectrum

The CM addresses the challenges of translational and rotational invariance in describing chemical systems by representing the system through pairwise Coulomb interactions. These interactions depend only on the relative distances between atoms, which remain invariant under rotations and translations of the chemical system. Equation 3 defines the construction of the CM (*M*_*ij*_)­

3 $$M_{\mathrm{ij}}=\{\begin{array}{l} 0.5Z^{2.4},\mathrm{for}\;i=j\\ \frac{Z_{i}Z_{j}}{\left|R_{i}-R_{j}\right|},\mathrm{for}\;i\neq j \end{array}$$

where the nondiagonal entries (*i* ≠ *j*) capture the Coulomb repulsion for all possible atom pairs, and the diagonal elements (*i* = *j*) approximate the potential energy of the isolated atoms. Although solving the spatial invariance problem, the CM still depends on the ordering of the atoms for the matrix. For overcoming this limitation, the eigenspectrum approximation is used, which consists of matrix eigenvalues ordered in a decrescent way. As the matrix eigenvalues do not depend on the rows or columns orderings, sorting the eigenvalues allows permutational invariance with a small loss of information.^44^

Through the eigenspectrum approximation that is built in the DScribe package,^62^ the CM may be utilized in regression tasks, presenting a good functionalization capability in numerous works on nanocluster systems sampling.^18–21,63^ However, the representation remains constrained to systems with identical atom counts, as more or less atoms alter the matrix dimensions and consequently the number of eigenvalues produced. To standardize feature vector lengths across systems, shorter eigenspectra are right-padded with zeros until they match the length of the eigenspectra corresponding to the largest system, ensuring a consistent dimensionality for all inputs. In our data set, the eigenspectrum length normalization is used mainly due to different adsorbate species (with different sizes) and nanoclusters that may have a smaller number of atoms than the site_size parameter. The CM eigenspectrum is a simple descriptor that does not require any parameters to define how the featurization process occurs other than the highest number of atoms being featurized, which allows for normalizing all eigenspectra during the eigenspectrum creation. Using CM as our baseline structural descriptor, our aim was to compare the results with the more robust MBTR, described next in Section 2.2.4. Therefore, for CM, only the effect of the site_size parameter (*s*) was assessed from *s* = 1 to *s* = 30. For fitting the train set and making predictions on the test set, the LinearRegressor algorithm from the Scikit-learn ML Python package^64^ was used.

#### 2.2.4 Many-Body Tensor Representation

MBTR encodes atomic environments using geometry-based functions: pairwise distances for *k* = 2 and angular relationships for *k* = 3, evaluated across all atomic combinations in a system, and grouped into classes based on element types.^45^ Each interaction class is represented on a discretized grid, where the outputs of the geometry functions (distances and angles) are broadened using Gaussian kernels. As implemented in the DScribe package,^62^ MBTR requires user-defined parameters for the grid: minimum and maximum values (min, max), number of bins (*n*) and Gaussian width (sigma). These parameters are shared across all classes, ensuring consistent feature vector dimensions across systems with varying atom counts. Hence, as shown in Figure 4, MBTR is a kernelized representation of the relative frequency of different many-body interactions organized into chemical classes.

**Figure 4:**
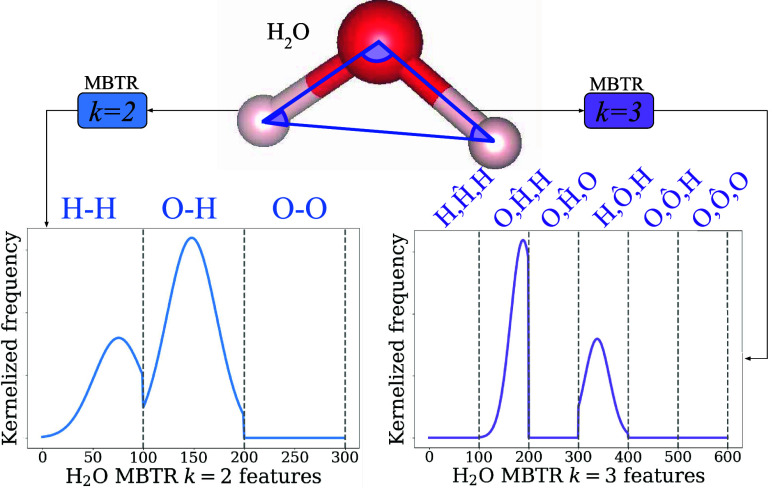
Water molecule featurization process for MBTR using distances (*k* = 2) and cosine angles (*k* = 3). The output flattened feature vector is viewed as a fixed Gaussian kernel density estimation of the geometry function outputs for all atoms, organized into consistently defined classes across a set with only H and O species.

However, MBTR’s feature vector size (and thus its computational cost) increases rapidly with chemical diversity as the number of interaction classes grows combinatorially. To manage this, the resolution of the grid is often reduced, potentially limiting the expressiveness of the descriptors. A key limitation is that MBTR requires all chemical species to be defined in advance, as it cannot featurize systems containing previously unseen elements, as interactions for these new species were not accounted for in the creation of the consistently ordered dimensions.

The localized variant, LMBTR, addresses this by introducing a dummy species *X* (*Z* = 0), restricting class combinations to those involving preselected atomic centers (X_c_). This localization reduces the number of interaction classes and the size of the resulting feature vector, which grows linearly with the number of declared centers. To consistently model adsorbed systems with respect to these centers, our implementation introduces the points argument as a setting to define the position of the LMBTR dummy species *X*. The user can select among several initialization options, such as

1. molC: geometric center of the adsorbate species;
2. molA: each adsorbate atom is initialized as a center for the LMBTR;
3. siteC: geometric center of the nanocluster site atoms;
4. siteA: each site atom site is initialized as a center for the LMBTR;
5. inter: geometric mean (midpoint) between molC and siteC.

Both MBTR and LMBTR were paired with Scikit-learn’s Ridge regression model^64^ to mitigate overfitting due to the high dimensionality of the characteristics. Optimization involved tuning the grid parameters (min, max, *n*), Gaussian width (sigma), and for LMBTR, the number and positions of the reference centers (points). For *k* = 2, the distance function was used with a fixed min of 0, while max was varied. For *k* = 3, angle cosine was used with a fixed grid spanning [−1, 1]. Table 1 lists the (L)­MBTR parameters explored. The number of site atoms (*s*) was also independently optimized. Full details of the parameter combinations are provided in the SI.

**Table 1:** Selected (L)­MBTR Parameters for Optimization

Parameter	Description
gridMax	Distance term grid maximum
*n*	Kernelization grid resolution
sigma	Gaussian kernel width
points	LMBTR X_c_ coordinates

### 2.3 Random Forest Regressor Optimization

Following the establishment of optimal featurization procedures for both the CM and LMBTR, these descriptors were utilized in conjunction with the RandomForestRegressor algorithm^46^ from the Scikit-learn library.^64^ Considering the intricate nature of the RFR model and the substantial dimensionality inherent in the LMBTR descriptor, a principal component analysis^65^ (PCA) was conducted to reduce the dimensionality of its feature set, thereby rendering it computationally feasible for multiple iterations of RFR optimization.

The Optuna hyperparameter optimization framework^58^ was used to carefully adjust the RFR hyperparameters within the specified ranges delineated in Table 2. Given the substantial quantity of LMBTR features, an RFR model with reduced tree depth, a decreased number of features, and more stringent leaf convergence criteria has been observed to achieve optimal performance. In the RFR optimization process using each descriptor, a consistent and randomized partitioning of the data set was implemented for all executions, with 80% used for training, 10% for validation, and 10% designated as a test set for the evaluation of the final optimized models.

**Table 2:** RFR Hyperparameter Optimization Ranges for Each Descriptor

RFR Parameter	CM	LMBTR
*n*_estimators	20–200	20–200
max_depth	10–150	10–50
max_features	1.0	0.1–1.0
min_samples_split	2	2–10

### 2.4 Generation of the External Test Set

Subsequent to the refinement of the descriptors and optimization of the algorithm, the machine learning model was evaluated through an independently generated external test set. The purpose of this process was to evaluate the transferability of the model, in particular its ability to accurately predict Δ*E*_tot_ values for instances absent from the initial data set. To achieve this, the nanocluster with the highest representation in the data set, identified as the Cu_13_ system, was chosen, as well as four adsorbate species: CO, CH_3_, CH_4_, and CH_3_OH. These adsorbed configurations correspond to approximately 4000 to 10,000 samples that involve the Cu_13_ nanocluster of the original data set. Furthermore, we incorporate the water molecule (H_2_O), which is present in the original data set solely as an adsorbate for oxide nanoclusters (and therefore not adsorbed on the Cu_13_ nanocluster), as a novel case for adsorption.

Adsorbed configurations were generated systematically using cluster_adsorption software,^66^ which automatically places the fixed adsorbate species at a specified distance above the static nanocluster surface, incorporating variations detailed in 0.1 Å, to produce numerous adsorbed configurations. To ensure consistency with the distance distribution of the adsorbate observed in the initial data set, the distances of the adsorbate groups 4 were uniformly selected, taking into account the first and third quartiles of the distance distributions shown in Figure 5.

**Figure 5:**
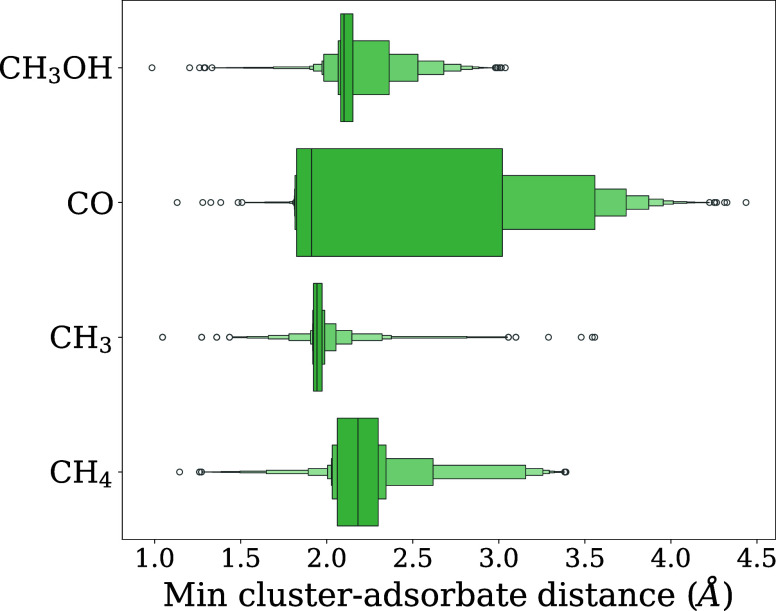
Distribution of adsorbate-nanocluster distances in data set for the selected adsorbates with the Cu_13_ nanocluster.

For each distance selected, 2000 configurations were generated, totaling 8.000 configurations per adsorbed system. To select representative structures from this large pool of results, the *k*-means clustering algorithm^67^ was used with the representation of the CM eigenspectrum as a similarity metric.^18–21^ By grouping each of the 2.000 generated configurations per adsorbate into 50 groups (*k* = 50), the structures closest to the centroid of each group were selected as the most representative.

In the case of the CH_3_ molecule, both its planar (gas phase ground-state) and pyramidal (py–CH_3_) structure (reflecting its geometry upon adsorption) were considered. Both followed the same generation methodology, but 25 configurations were sampled for each, ensuring that the final set for this molecule comprised 50 representative adsorbed structures, as in the other molecules. Single-point DFT calculations were performed to obtain the relative total energy of the structures selected for this external test set.

As a final experiment of modeling the adsorbed systems with the optimized structural descriptors, we included an additional electronic feature to the CM as an additional descriptor to improve model performance on the external test set. This feature is the total number of unpaired electrons in the adsorbate, defined as a single digit for each data set sample. For employing this feature, we simply concatenate it to the optimized CM feature vector and retrain the random forest regressor with the same optimized hyperparameters as for the CM.

### 2.5 Total Energy Calculations

Our total energy calculations for both the data set and the external test set were performed using the spin-polarized DFT framework, employing the semilocal exchange-correlation energy functional proposed by Perdew, Burke and Ernzenhof (PBE).^68^ Given the diverse energy regimes exhibited by adsorbed systems, ranging from physisorption to chemisorption, it is crucial to account for dispersion interactions, particularly in systems characterized by weak bonding. Thus, to improve the accuracy of plain DFT-PBE calculations, we have integrated van der Waals corrections as proposed by Tkatchenko and Scheffler^69^ for the DFT-PBE total energy.

The resolution of the Kohn–Sham (KS) equations for the cluster models was executed via the Fritz–Haber Institute materials simulations (FHI-aims) package,^60,61^ wherein the KS orbitals were expanded into numerical atom-centered orbitals,^61^ beginning from the minimal basis set (representing free atoms) and extending to the second tier of basis set refinement (*light tier2*). Electrons were addressed using the scalar relativistic framework within the atomic zeroth-order relativistic approximation (atomic ZORA).^70^ A Gaussian broadening parameter of 10 meV was used to guarantee the appropriate occupancy of the electronic states proximal to the highest occupied molecular orbital. Calculations pertaining to the external test set were carried out as single-point evaluations (excluding geometry optimization) to facilitate direct comparisons with ML model predictions.

## 3 Results and Discussion

In this section, we present and analyze the results obtained throughout the development of the model and its final evaluation on new data. We begin in Section 3.1 by exploring the site size and (L)­MBTR parameters to identify the optimal performance protocols for our regression problem. Next, we assess the performance of an optimized RFR implemented with the optimal set of features for both CM and LMBTR (Section 3.2). Finally, in Section 3.3, we conduct a comprehensive evaluation of the models to validate their predictive capabilities on newly generated data, our external test set, outlining its limitations and perspectives.

### 3.1 Structural Descriptors Linear Optimization

The results of the optimization of the size of the CM eigenspectrum site (*s*) with linear regression are presented in Figure 6. Based on the observed MAE values, ranging from 0.875 to 1.075 eV, the inclusion of more than 9 site atoms in the CM representation does not lead to significant improvements in predictive performance. Although increasing the number of atoms improves the representation of larger nanoclusters, such as Cu_55_ and Cu_42_Zn_13_ other oxide nanoclusters, it results in numerous dummy features for smaller nanoclusters of 13 or fewer atoms, which constitute most of the data set. Given the initial goal of locally modeling the adsorption sites, and the lower MAE obtained, a site_size of *s* = 9 atoms was selected as the optimal configuration for the CM representation.

**Figure 6:**
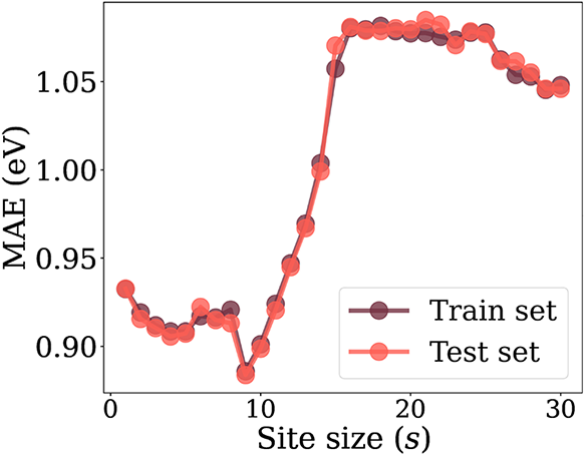
Linear regression results for the CM eigenspectrum with different site sizes (*s*).

Unlike the CM eigenspectra, which depend solely on the site size parameter to model the adsorbed systems, the MBTR involves several user-defined parameters, as listed in Table 1. Its local variant, LMBTR, additionally requires specifying the coordinates used to initialize the kernelization centers, which in our implementation are described in Section 2.2 as molC, molA, siteC, siteA, and inter. Such parameters can be applied to the two-body (*k* = 2) or three-body (*k* = 3) terms, defining how the (L)­MBTR kernelization is performed for each case. For each combination of these parameters, one must featurize all Cartesian coordinates in our large data set, which consumes significant computational power. With the data set that has been made, the linear ridge regression algorithm^71^ is trained and its performance assessed in the test set, from which a MAE is extracted.

Hence, due to the prohibitive computational costs posed by the large sample size, the optimization of the (L)­MBTR parameter was performed manually through a guided exploration of the parameter space, balancing descriptor performance and computational efficiency. The results of this optimization are presented in Section 3.1.1, where the effect of each parameter is analyzed, either on computational cost or descriptor performance. Section 3.1.2 presents the final treatment of the LMBTR feature vector with the optimal settings selected through PCA, effectively reducing the computational burden associated with using this descriptor on our large data set.

#### 3.1.1 MBTR Parameter Optimization

To assess the impact of site size and (L)­MBTR parameters on the test set MAE, we systematically explored a wide range of combinations of MBTR and LMBTR settings, resulting in a total of 657 distinct tests. All results were analyzed and the most important findings are shown in Figures 7 and 8 for the terms two-body (*k* = 2) and three-body (*k* = 3), respectively, where we plot the individual tests with the MAE achieved in relation to each optimized parameter.

**Figure 7:**
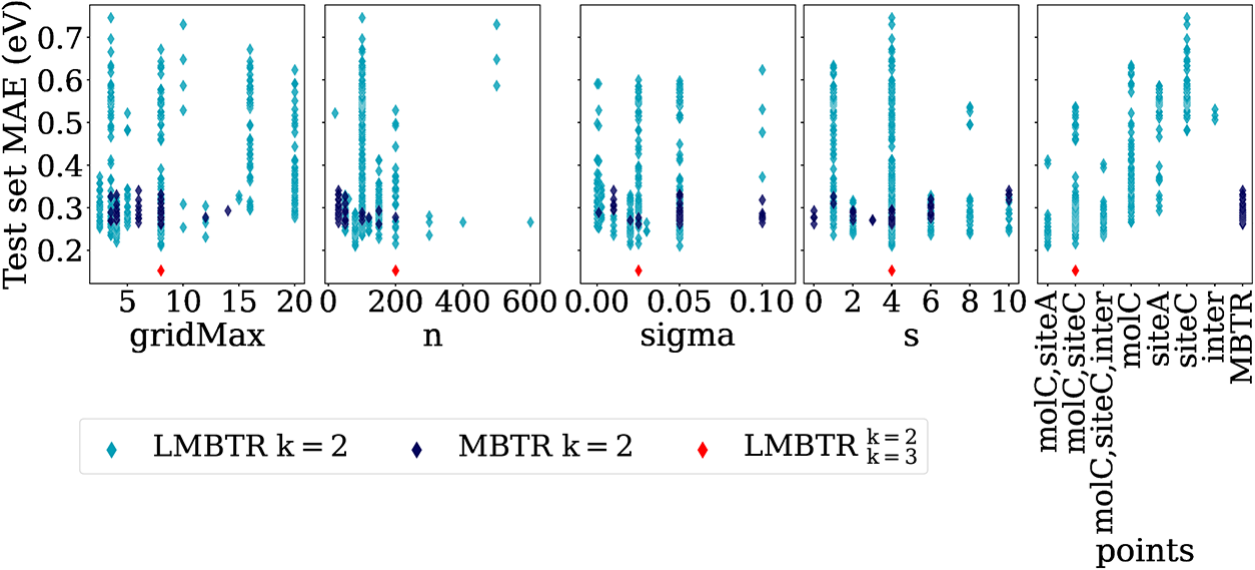
Scatter plot of test set MAE results from all executions using the ridge regressor and (L)­MBTR *k* = 2 parameters. Each point corresponds to a unique combination of *k* = 2 parameters shown on the *x*-axis: gridMax (upper bound of the kernelization grid), *n* (grid discretization), sigma (Gaussian broadening), *s* (site size), and points (centers coordinates for LMBTR). The result obtained by concatenating the selected *k* = 2 and *k* = 3 parameters is highlighted in red.

**Figure 8:**
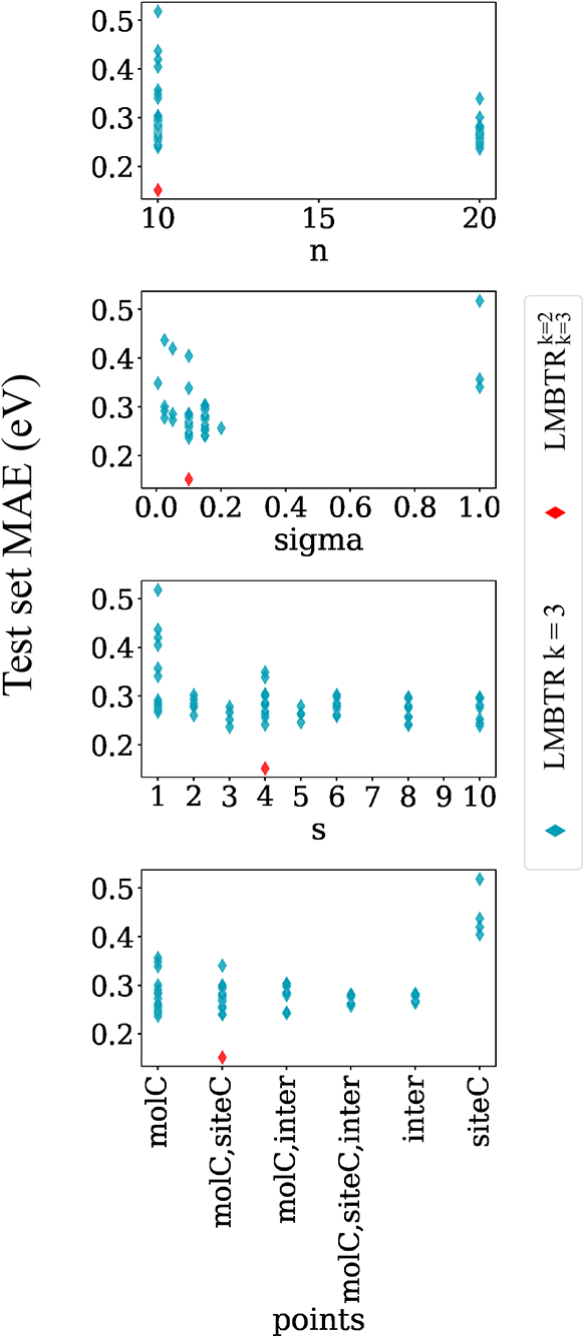
Scatter plot of test set MAE results from all executions using the ridge regressor and (L)­MBTR *k* = 3 parameters. Each point corresponds to a unique combination of *k* = 2 parameters shown on the *x*-axis: *n* (grid discretization), sigma (Gaussian broadening), *s* (site size), and points (centers coordinates for LMBTR). The result obtained by concatenating the selected *k* = 3 and *k* = 2 parameters is highlighted in red.

The high chemical diversity of the data set, which directly impacts the dimensionality of the (L)­MBTR feature vectors, limited the number of parameter combinations that could be explored for the MBTR. This descriptor requires initializing the product of combinations (excluding symmetric terms) of pairs and triplets between all possible atomic species encountered in the data set, while the LMBTR restricts the total number of combinations only by accounting for groupings with the pseudospecies X, which is initialized at any number of given spatial coordinates to localize the representation. For our data set, at a reasonable resolution for accurately discerning kernels (*n* = 100), the total number of grid points for each combination of species may account for tens of thousands of features depending on the geometry function, the use of MBTR or LMBTR, and the number of LMBTR centers. Specifically, the *k* = 3 term further increases this cost due to the combinatorial growth of three-body terms, making it feasible for the LMBTR only at reduced grid resolutions (*n* = 10 or *n* = 20).

For the *k* = 2 term (Figure 7), LMBTR consistently outperformed the resource-limited MBTR that yielded consistent MAE values around 0.3 eV in all parameter variations. The MBTR initialization of the centers at all atoms may include redundant information from regions outside the adsorption site, whereas the LMBTR enables a more selective and higher resolution characterization of the adsorption region. Among the parameters tested, most did not show clear trends or convergence toward a lower MAE, except for the points argument, which determines where the LMBTR centers are initialized. Including the molecular geometric center (molC) proved essential as its absence consistently resulted in worse performance in all cases. Including a single center on the site atoms (siteC) also did not show better results than molC, however declaring centers in all the site atoms (siteA) showed better results. The lowest MAEs are achieved by combining molC with siteA (molC, siteA), however marginally improving accuracy at the order of 0.01 eV in relation to the combination molC,siteC. The inter center, localized in between siteC and molC, did not improve the performance of the model, even when combined with molC and siteC (molC,siteC,inter). Hence, due to time and memory constraints, molC,siteC was selected as the optimal parameter for the LMBTR centers.

Regarding the site_atoms parameter, selecting *s* = 4 yielded the lowest MAE in preliminary tests and was maintained in subsequent experiments, as four atoms typically capture the relevant local environment for adsorption. Moreover, this setting reduces the computational burden of LMBTR, especially in cases where a larger number of centers are declared, such as site A. In terms of grid resolution (*n*), MAE plateaued at *n* = 200, suggesting that further increases in discretization do not enhance model performance. The size of the kernel grid (gridMax) was not critical, but the width of the kernel (sigma) showed a clear convergence around sigma = 0.05, indicating the optimal Gaussian kernel width to minimize information loss due to superposition between different Gaussians.

Divergent behavior was detected for the *k* = 3 LMBTR term depicted in Figure 8. The parameters site_size (*s*) and points demonstrated a lower impact on minimizing MAE. In contrast, elevating the grid resolution from *n* = 10 to *n* = 20 resulted in a notable enhancement in MAE, with further improvements expected at higher resolutions. The sigma parameter assumed greater significance due to coarser resolution, necessitating optimal kernel width to accurately capture angular features. To maintain consistency, the final configurations for the LMBTR terms *k* = 2 and *k* = 3 were set at *s* = 4 and points = molC, siteC, respectively.

Based on the MAE values of the test set, both the MBTR and LMBTR descriptors outperform the Coulomb matrix when used with linear regression methods. A substantial reduction in the best MAE is observed from 0.85 eV with CM to 0.26 eV for the MBTR^*k*=2^, 0.23 eV for the LMBTR^*k*=2^, and 0.21 eV for the LMBTR^*k*=3^ descriptors. The term *k* = 3 slightly outperformed *k* = 2 even at a much smaller resolution (*n* = 10 versus *n* = 200), showing the importance of nanocluster adsorbate angles in the prediction of Δ*E*_tot_. When the *k* = 2 and *k* = 3 descriptors were concatenated into a single feature vector, the regression performance improved further, reducing MAE to 0.17 eV.

#### 3.1.2 Final LMBTR Feature Vector

Upon determining the optimal settings for LMBTR, presented in Table 3, the final descriptor was established by concatenating the feature vectors *k* = 2 and *k* = 3. The term *k* = 2, employing a grid resolution of *n* = 100, yielded 9.000 features, while the term *k* = 3, configured with a grid resolution *n* = 10, produced 9.500 features. This resulted in a cumulative total of 18.500 features for the concatenated LMBTR descriptor. Taking into account the inherent high dimensionality, PCA was performed to reduce the computational cost of performing executions with this large feature vector by reducing its dimensionality, as shown in Figure 9.

**Figure 9:**
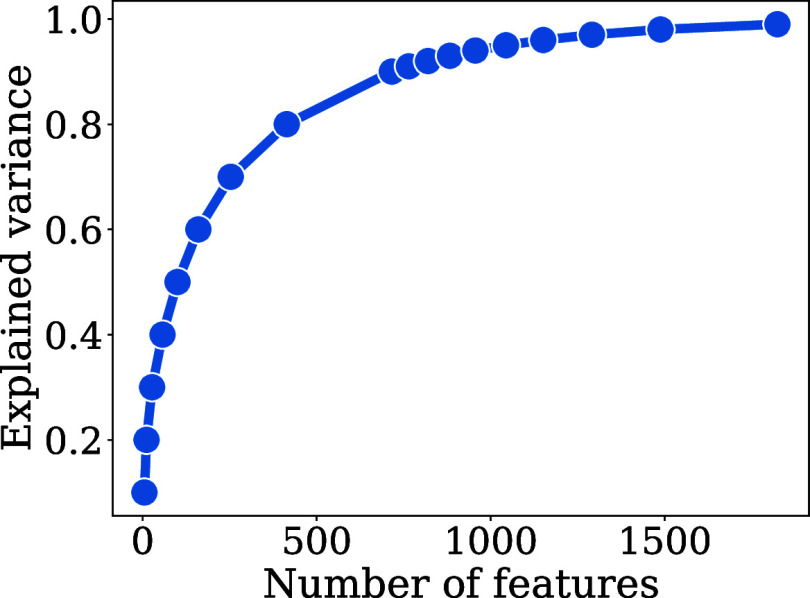
Principal component analysis results for the concatenated *k* = 2, *k* = 3 LMBTR.

**Table 3:** Best LMBTR Parameters for *k* = 2 and *k* = 3 Terms from the Linear Optimization, Both with *s* = 4

MAE (eV)	geometry	min	max	*n*	sigma	scale	points
0.235	distance	0	3.5	100	0.05	0.25	molC, siteC
0.209	cosine	–1	1	10	0.1	0	molC, siteC

The high sparsity of the final LMBTR feature vector is observed from the great reduction in the total number of features of 18.500 to the 1.800 principal components that explain 99% of the total variance in this set of features. This is mainly due to the class-oriented nature of the LMBTR descriptors, which organizes the encoded structural information into classes of two and three body terms. Hence, for any given sample, only classes with species present in the adsorbed system will have information, whereas absent species combinations result in zero entries. The principal components obtained linearly transform the LMBTR classes in directions that capture the greatest variance in the data set, effectively reflecting how all classes interact and distinguishing adsorbed systems based on these components. Since the resulting 1.800 features constituted a manageable number of available computational resources throughout the modeling pipeline, this PCA transformation was selected to define the final LMBTR feature vector for our model. The final concatenated and PCA transformed LMBTR vector was tested using the ridge regression, as shown in Figure 10, with the best parameters for both *k* = 2 and *k* = 3 LMBTR terms summarized in Table 3.

**Figure 10:**
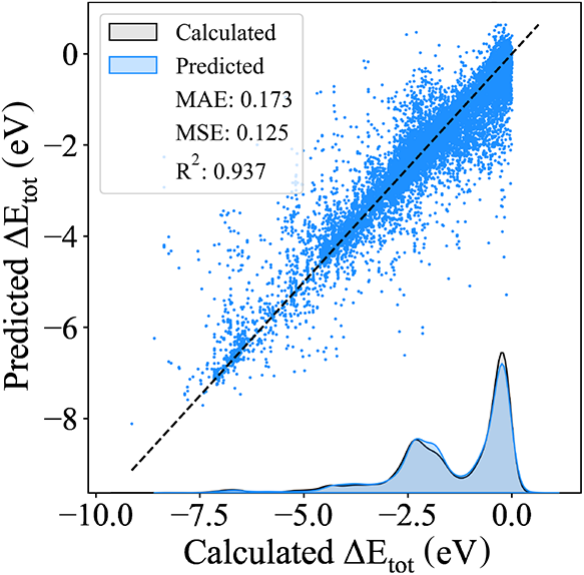
Ridge regression results for the PCA transformed concatenated *k* = 2, *k* = 3 LMBTR feature vector.

### 3.2 Random Forest Regressor Model

The optimal hyperparameters obtained with Optuna for the RFR using each descriptor are summarized in Table 4. Both models achieved comparable performance on the test set, with MAE around 0.05 eV. The fraction of features used per tree (max_features) remained similar for both descriptors, approximately 60%. However, key differences emerged in other hyperparameters, particularly the tree depth (max_depth), which appears to correlate with the number of features in each descriptor. The CM descriptor, consisting of fewer than 20 features, required a much deeper tree (max_depth= 78), whereas the LMBTR descriptor, with roughly 1.800 features, achieved optimal performance with a shallower depth (max_depth = 31). This contrast suggests that the CM benefits from deeper trees to capture feature interactions, while the high-dimensional LMBTR relies more heavily on feature sampling and regularization strategies to prevent overfitting.

**Table 4:** Optimal RFR Hyperparameters for Each Descriptors with Test Set MAEs

RFR Parameter	CM	LMBTR
*n*_estimators	100	136
max_depth	78	31
max_features	0.64	0.67
min_samples_split	8	8
MAE	0.048 eV	0.054 eV

Further differences were observed in the number of estimators, with LMBTR requiring more trees ( *n*_estimators = 136) compared to CM ( *n*_estimators = 100), likely to manage its increased complexity of features. In contrast, min_samples_split and min_samples_leaf remained similar across both descriptors, serving primarily as a regularization role to mitigate overfitting, relative to the number of data samples, regardless of descriptor complexity. These findings underscore how the descriptor dimensionality influences hyperparameter optimization in RFR models and emphasize the importance of hyperparameter tuning for model performance.

The prediction distributions for both descriptors are shown in Figure 11. Both models achieved MAEs lower than the expected DFT error of 0.1 eV for adsorbed systems.^47–49^ None of the descriptors exhibited systematic bias toward over or underestimating Δ*E*_tot_, and only a few outliers were present. The final MAEs obtained were 0.048 eV for the CM and 0.055 eV for the LMBTR. Although the simpler CM performed significantly worse than the LMBTR in linear regression, the use of the RFR effectively equalized the performance of the two descriptors.

**Figure 11:**
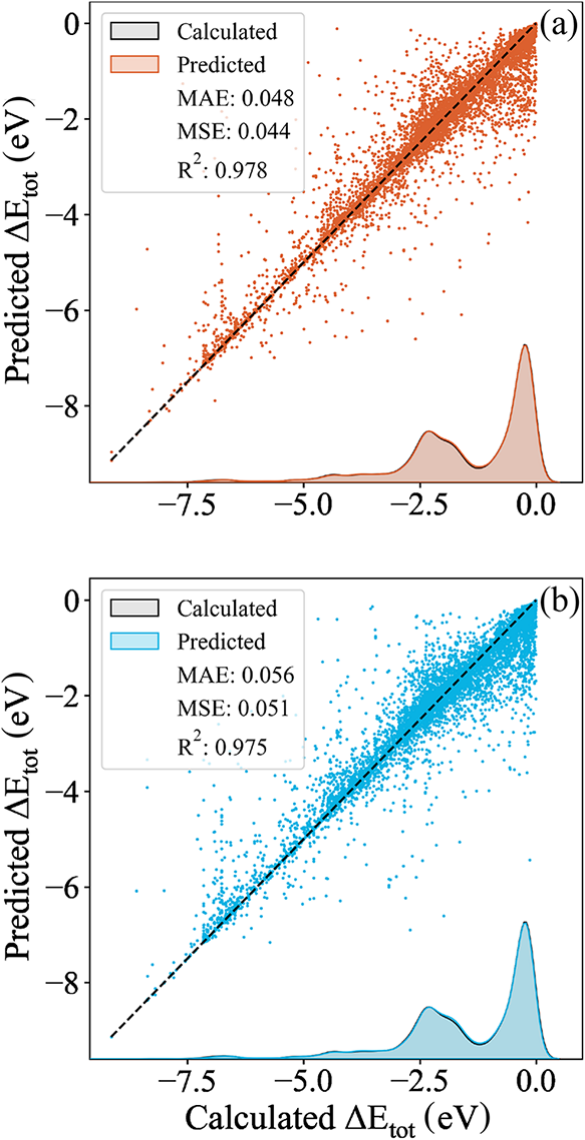
Prediction distribution on the test set using the optimized random forest regressor for the two optimized structural descriptors: (a) CM eigenspectrum computed with 9 site atoms, and (b) Concatenated LMBTR with *k* = 2 and *k* = 3 terms, computed with 4 site atoms.

In Figure 12, the MAE is presented in relation to different data set categories: (a) nanocluster compositions, (b) nanocluster sizes, and (c) adsorbate species. The MAEs for both the Coulomb matrix (CM) and LMBTR descriptors are displayed alongside the total number of samples in the data set for each category. Overall, the predictive performance of both descriptors using RFR is similar, with large deviations in MAE observed primarily in underrepresented categories. For example, nanoclusters based on Ru, as well as those with 55 atoms (notably Cu_42_Zn_13_ and Cu_55_), show significantly higher MAEs, reaching up to 0.35 eV.

**Figure 12:**
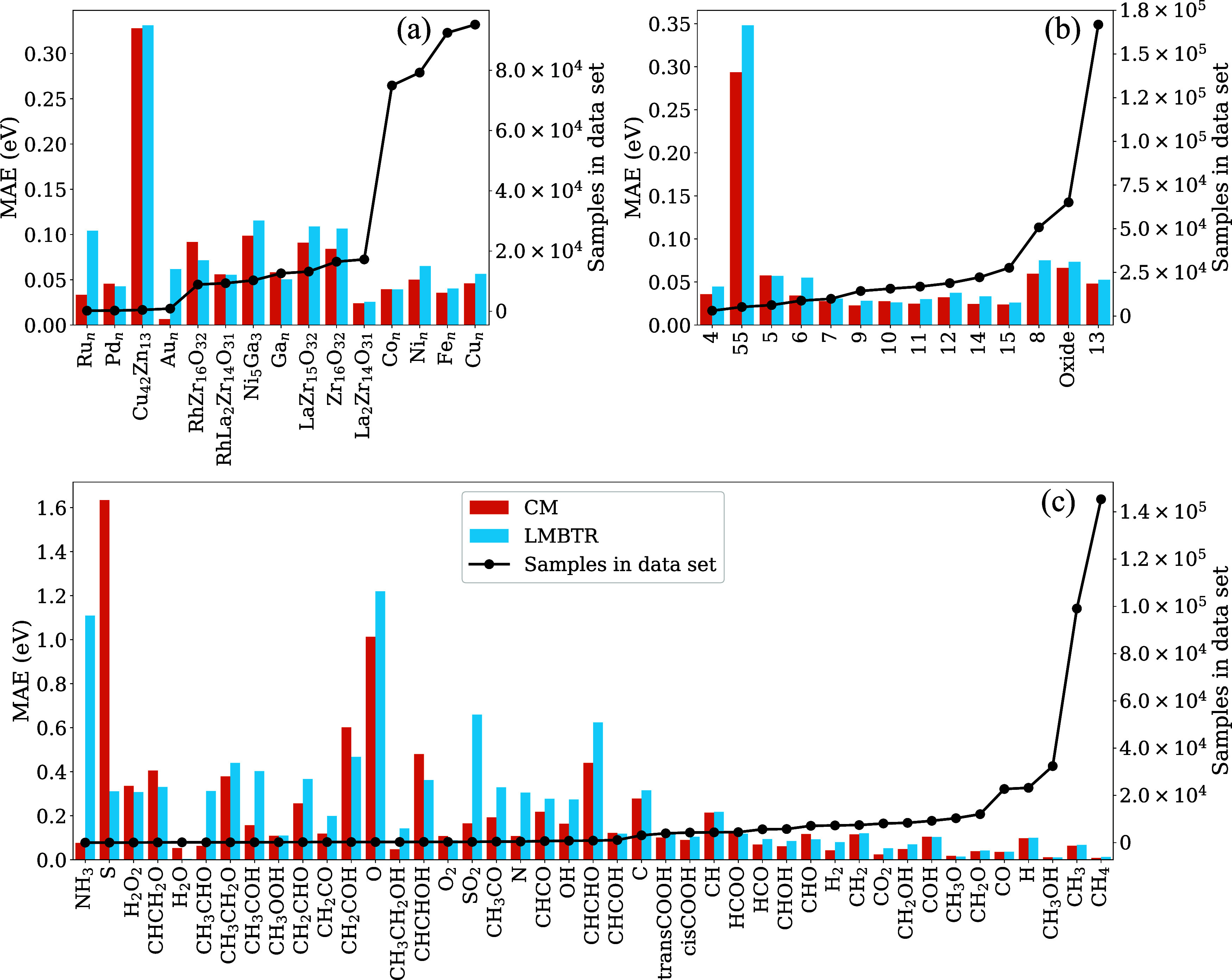
MAEs and total sample count in the data set for both descriptors with the RFR grouped by (a) nanocluster compositions, (b) nanocluster sizes, and (c) adsorbate species.

The influence of sample representativity is particularly pronounced in the categories of adsorbate species. As shown in panel (c), adsorbates such as NH_3_, S, and O exhibit MAEs exceeding 1.0 eV, far above the general trend. In contrast, adsorbate categories with larger sample counts (above 4.000) consistently yield MAEs below 0.15 eV, highlighting a strong correlation between data availability and model precision. This suggests that improving the uniformity of the data set, particularly among adsorbates, could significantly enhance the descriptor performance and generalizability of the model.

### 3.3 Final Model Assessment

In order to assess the model’s capability, specifically its capacity to accurately predict Δ*E*_tot_ values for adsorbed configurations that have not been previously observed, an external test set was constructed. This set was autonomously generated to maintain a distance distribution similar to that of the original data set. For each selected adsorbed system, 2.000 configurations were randomly generated and subsequently refined down to the most representative configurations 50 applying the clustering algorithm *k*-means.^18–21,67^ The most prevalent nanocluster identified within the data set (Cu_13_) was chosen as the adsorbent and combined with adsorbates CH_3_ (both in planar and pyramidal forms), CH_4_, CO, and CH_3_OH. In addition, the H_2_O molecule, absent from any combination with the Cu_13_ nanocluster in the original data set, was included to explore the model’s ability to transfer acquired knowledge to novel combinations.

#### 3.3.1 Descriptor Comparison on the External Test Set

In Figure 13, the box plots illustrate the prediction errors for the selected adsorbates within the external test set, analyzed using two different descriptors with the RFR model. Regarding the CH_4_ molecule, both descriptors demonstrated satisfactory performance, despite the presence of outliers with significant underestimations in the CM descriptor. The CH_3_OH molecule achieved the highest accuracy, as evidenced by the nearly negligible errors in both models. In contrast, for adsorbates CH_3_, py–CH_3_, and CO, there were more marked discrepancies between the two descriptors.

**Figure 13:**
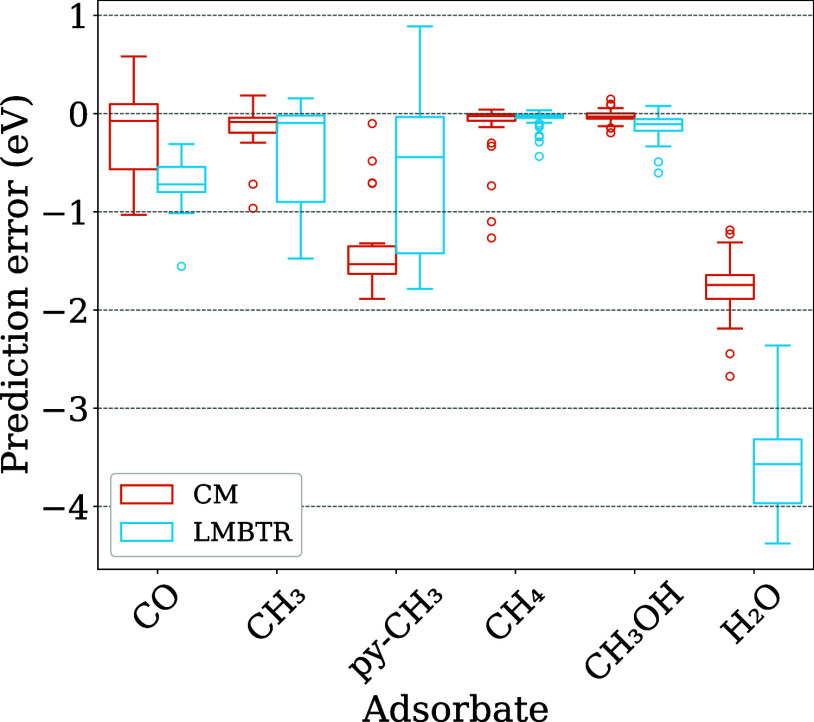
Box plot for the external test set prediction errors for each adsorbate with the Cu_13_ nanocluster using the optimized RFR with each descriptor.

In addition to the case that was not in the original data set (H_2_O adsorbed on Cu_13_) the most pronounced discrepancies were observed for the CH_3_ molecule. For the pyramidal (py-CH_3_) geometry, LMBTR achieved errors below 1.0 eV in approximately 50% percent of its predictions, while the CM consistently exhibited errors exceeding 1.5 eV. In contrast, for the planar CH_3_, the CM descriptor demonstrated only two outliers with errors reaching up to 1.0 eV, whereas the LMBTR showed errors that exceeded 0.5 eV for almost 50% percent of its predictions. These results emphasize a significant sensitivity of the CM descriptor to geometric variations in isomeric adsorbates such as CH_3_ and py–CH_3_, a phenomenon also seen, though less significantly, in LMBTR.

Furthermore, the influence of the distance between the adsorbate and nanocluster on the performance of the model was assessed, as shown in Figure 14. Overall, the minimum distance between the adsorbate and the nanocluster did not correlate strongly with the prediction accuracy. However, for the CO molecule, CM performed significantly better at larger distances compared to LMBTR. Some outliers appeared at closer or farther distances for the CM, while the LMBTR did not show a clear dependence of the error on distance.

**Figure 14:**
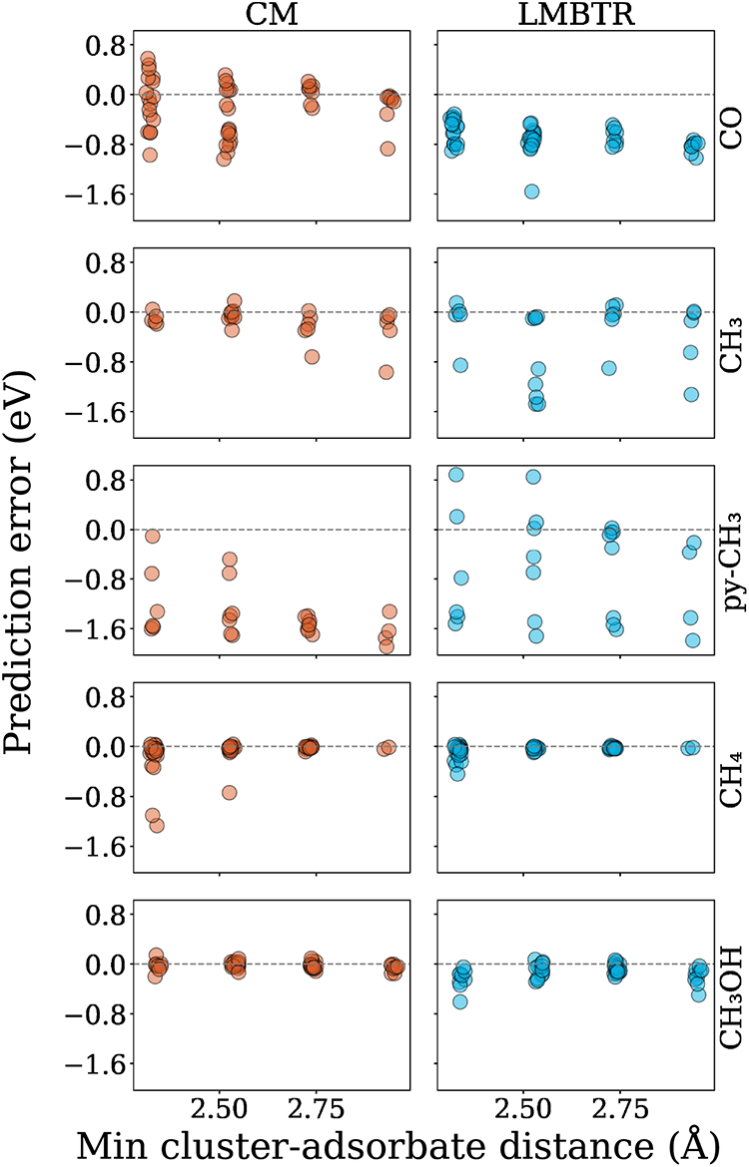
External test set errors in relation to the minimum nanocluster-adsorbate distance for both descriptors with the RFR.

#### 3.3.2 Unpaired Electrons as Feature

Although both models performed similarly on the internal train and test sets with MAEs of 0.02 eV (train) and 0.05 eV (test), performance degraded considerably on the external test set, although it was composed of the most representative adsorbent, Cu_13_. In particular, predictions for H_2_O/Cu_13_ system, which was the only one that was not presented in the original data set, yielded consistently high errors, with neither structural descriptor achieving errors below 1.0 eV. From a chemical point of view, a very important knowledge that affects whether a species will bind weakly or strongly is the presence of unpaired electrons in the molecule—closed shell species tend to bind weakly, whereas radicals with unpaired electrons tend to bind more strongly. However, this information is not present in any of the descriptors which are structural and do not include electronic information.

As depicted in Figure 15, adding the number of unpaired electrons from the adsorbate species to the CM feature vector allows correction of the predictions for the H_2_O molecule. This effect is very significant, approximating otherwise poor predictions from the CM or LMBTR to the typical DFT error of 0.1 eV,^47–49^ in relation to experimental results on adsorption energies. However, the proposed electronic feature did not improve the predictions for other adsorbates than H_2_O molecule, and we note key differences between the CM and LMBTR structural descriptors, which performed equally well at a MAE of 0.05 eV in the internal test set.

**Figure 15:**
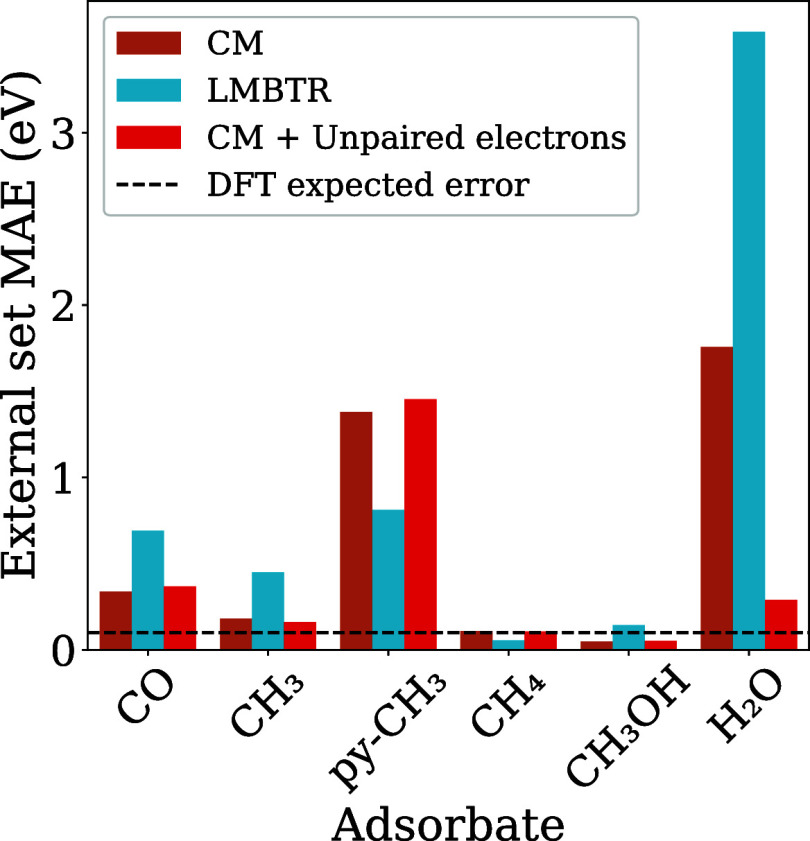
Predictions MAEs for each adsorbate with the Cu_13_ nanocluster in the external test set using the optimized RFR and descriptors.

With the exception of the adsorbates CH_4_ and CH_3_OH, the remaining adsorbates exhibited significant variations in the MAE for either structural descriptor. As noted, for molecules CO and CH_3_ the CM has closer MAEs to the expected DFT error than LMBTR. However, the pyramidal py-CH_3_ adsorbate, which showed the highest errors as one of the most recurrent systems with Cu_13_ in the data set, has a lower MAE with LMBTR. Although the pyramidal configuration of the CH_3_ molecule characterizes its mode of chemisorption, the distances used to construct the adsorbed configurations may diverge from the actual adsorption distances, leading to higher observed errors due to chemical configurations not present in the data set for this molecule. The inclusion of the electronic feature did not improve the predictions for this adsorbate, showing that the accuracy remains contingent upon the representativeness of the sample.

## 4 Conclusions

Although numerous studies in the literature have documented outstanding performance of machine learning models that deploy structural descriptors,^40^ these efforts are generally confined to a limited range of adsorbed systems. Although more advanced conceptualizations of molecular descriptors have exhibited incremental improvements–frequently utilizing the CM as a foundational benchmark for comparison^24^—our findings indicate that analogous predictive precision can be achieved through the use of adequately sophisticated regression algorithms, such as RFR, with the CM even surpassing the LMBTR in performance on the external test set.

However, structural descriptors alone are insufficient for developing robust and transferable models capable of generalizing across a wide range of nanocluster structures, compositions, and adsorbates. Although low prediction errors—below the expected DFT uncertainty of 0.1 eV^47–49^ in relation to experimental results—can be achieved for well-represented adsorbates (approximately at four thousand adsorbed configurations), the model fails to extrapolate to underrepresented or novel cases. These findings highlight that the diversity and quantity of training data are much more critical to model performance than the choice of descriptor or regression algorithm. Ultimately, the model lacks the ability to learn the fundamental physicochemical principles that govern adsorption only from structural information, which limits its transferability.

Our analysis reveals that the consistency of predictions within the data set remains unobservable for newly generated adsorbed configurations, even within the most frequently represented system. This observation raises concerns regarding the feasibility of applying such models to particular adsorbed systems, given the substantial computational expense entailed in generating DFT-derived training data. To address these constraints, future investigations should prioritize hybrid featurization strategies that integrate physicochemical insights to more accurately capture adsorption phenomena. In addition, active learning methodologies can present a promising avenue by facilitating the selection of the most informative data points, thus minimizing the required number of DFT calculations while preserving predictive efficacy for screening purposes.

## Supplementary Material



## Data Availability

As mentioned, all DFT calculations were done using the FHI-aims package, which can be used under a nonfree academic license. Additional details can be obtained from the link https://fhi-aims.org/. The developed code, processed data, and models can be encountered in https://github.com/QTNano/cluster-mol-predictor and 10.5281/zenodo.17027471. Furthermore, additional details are provided within the electronic Supporting Information, while additional crude data can be obtained directly with the authors upon request.

## Notes

Data used for the figures, as well as complementary analyses and additional technical details (PDF)
