# Supplementary material for: Optimizing Molecular Descriptors for Reliable Adsorption Energy Prediction on Transition Metal Nanoclusters
Source: ACS Omega. 2026 Jan 6;11(2):2962–75. doi: 10.1021/acsomega.5c09138 (PMC12824938; doi:10.1021/acsomega.5c09138)
Supplement: Supplementary file 1 [file ao5c09138_si_001.pdf]

Electronic Supporting Information File: Optimizing Molecular Descriptors for Reliable Adsorption Energy Prediction on Transition Metal Nanoclusters

Lucas B. Pena,[*,†] Felipe V. Calderan,[*,‡] Priscilla Felício-Sousa,[*,¶] Karla F. Andriani,[*,§] Marcos G. Quiles,[*,‡] Juarez L. F. Da Silva,[*,¶] and Breno R. L. Galvão[*,‖]

†*Centro Federal de Educação Tecnológica de Minas Gerais,* 30421-169, *Belo Horizonte, MG, Brazil*

‡*Institute of Science and Technology, Federal University of São Paulo,* 01109-010, *SP, Brazil*

¶*São Carlos Institute of Chemistry, University of São Paulo, Av. Trabalhador São-Carlense* 400, 13560-970, *São Carlos, SP, Brazil*

§*Departament of Exact Sciences, State University of Santa Cruz,* 45662-900, *Ilhéus, BA, Brazil*

‖*Centro Federal de Educação Tecnológica de Minas Gerais,* 30421-169, *Belo Horizonte, MG, Brazil*

E-mail: lucas.bernardesp205@gmail.com; fvcalderan@unifesp.br; priscillafelicio@me.com; karlandrianiqmc@gmail.com; quiles@unifesp.br; juarez_dasilva@iqsc.usp.br; brenogalvao@gmail.com

Contents

S-1 Introduction S-3

S-2 Additional Information on the Data set S-3

S-3 Additional Information on Descriptor Optimization S-8

S-4 Random Forest Regressor Detailed Results S-9

S-4.1 Coulomb Matrix in Data Set S-9

S-4.2 LMBTR in Data Set S-11

S-4.3 External Test Set Detailed Results S-13

S-5 Tables S-14

S-5.1 MBTR k=2 Optimization S-14

S-5.2 LMBTR k=2 Optimization S-15

S-5.3 LMBTR k=3 Optimization S-27

S-5.4 Optuna Hyperparameter Optimization S-29

S-5.4.1 RFR + CM Optimization S-29

S-5.4.2 RFR + LMBTR Optimization S-30

References S-33

S-1 INTRODUCTION

This document provides supplementary details supporting the main findings outlined in the manuscript. Detailed data are presented herein to facilitate the transparency and reproducibility of the reported findings. In this context, we systematically analyze the data set by enumerating the sources of all the DFT data employed, identifying the origin of each adsorbed system. In addition, we provide in-depth information concerning the descriptors utilized and the associated outcomes of the implemented random forest algorithms.

S-2 ADDITIONAL INFORMATION ON THE DATA SET

The data set used in this study is derived from FHI-aims[1] calculations performed on a wide range of molecular nanocluster-adsorbate systems,[2–9] as presented in Table S-1, that is, the final data set is made up of several small data sets with different characteristics. The output files originating from the FHI-aims geometry optimizations were systematically parsed to extract the total energy corresponding to each set of Cartesian coordinates obtained throughout the local optimization process. By incorporating frames from the geometry optimization process, the data set encapsulates not only the ultimate optimized geometry, but also intermediate, less stable configurations.

This data set comprises an aggregation of nine distinct and recent studies, with publication dates ranging from 2020 to 2024, all derived from calculations of high precision first principles using the FHI-aims code. In total, the collection encompasses more than $425,000$ unique data points or samples, serving as a significant and contemporary resource for the materials science and computational chemistry communities. The scale and quality of this dataset make it exceptionally well suited for the development of data-driven models to expedite the discovery and understanding of catalytic processes.

Table S-1. Description of the data set sources obtained by FHI-aims calculations and their substrates and adsorbates species, as well as the number of data extracted from the structure optimizations.

Reference Data Source	Nanocluster Substrates	Adsorbates Species	Samples
K. F. Andriani *et al.* 2	Fe_{13}, Co_{13}, Ni_{13}, Cu_{13}	H, C, CH, CH_2, CH_3, CH_4	8,551
P. Felicio-Sousa *et al.* 3	Fe_{13}, Co_{13}, Ni_{13}, Cu_{13}	H_2, CH_4, CO, CH_3OH	38,313
K. F. Andriani *et al.* 4	Fe_n, Co_n, Ni_n, Cu_n, $n = 4 - 15$	H, CH_3, CH_4	108,789
M. N. Collacique *et al.* 5	Fe_8, Co_8, Ni_8, Cu_8, Ru_8, Pd_8	CO_2	86,911
Rafael A. De Sousa *et al.* 6	Ni_8, Ga_8, Ni_5Ga_3	H, H_2, CO, HCO, CO_2, HCOO, CH_4	37,617
I. L. Gomes *et al.* 7	$Zr_{16}O_{32}$	H, H_2, H_2O, H_2O_2, C, CH, CH_2, CH_3, CH_4 CO, CO_2, O, O_2, OH, N, NH_3, S, SO_2, CH_3CH_2OH, CH_3OH, CH_3OOH	5,369
V. K. Ocampo-Restrepo *et al.* 8	Cu_{55}, $Cu_{42}Zn_{13}$	CH_3CO, CHCHO CHCOH, CH_2CO, CH_3CHO, CH_3COH, CH_2CHO, CH_2COH, CH_3CH_2O, CH_3CH_2O, $CHCH_2O$, CHCHOH	5,859
P. Felício-Sousa *et al.* 9	$Zr_{16}O_{32}$, $RhZr_{16}O_{31}$, $LaZr_{15}O_{31}$, $La_2Zr_{14}O_{31}$, $RhLa_2Zr_{14}O_{31}$	H, CH_3, CH_4	34,277
K. F. Andriani *et al.* (Publication in progress)	Fe_{13}, Co_{13}, Ni_{13}, Cu_{13}	CO, COH, CH_2O, CH_3O, CH_2OH, CH_3OH	100,238

The aggregated data primarily emphasize the chemistry C_1, which involves reactions of molecules that contain a single carbon atom. The consistent presence of adsorbates such as CO, CO_2, CH_4, and their various hydrogenation intermediates (CH, CH_2, CH_3, HCO, etc.) from multiple sources underscores a concerted research effort to address critical energy and environmental applications. These include the catalytic reduction of carbon dioxide to produce value-added chemicals such as methanol (CH_3OH), the activation of methane for industrial precursors and the conversion of syngas (a mixture

of CO and H_2). The addition of C_2 species (e.g. CH_3CH_2OH) in selected studies indicates a further expansion of this work towards the synthesis of more complex hydrocarbons, thus broadening the overall applicability of the data set.

The data set is further delineated by its extensive variety of nanocluster substrates, offering a broad chemical space for exploration. A substantial segment of the data investigates catalysis in pure transition metal nanoclusters, with a primary focus on first-row elements (Fe, Co, Ni, Cu), while also encompassing precious second-row metals (Ru, Pd). The size of these clusters is methodically varied, ranging from 4 to 55 atoms, which is pivotal for examining the effects of quantum size on catalytic activity. In addition to pure elements, the data set includes bimetallic and alloy systems, such as Ni_5Ga_3 and $Cu_{42}Zn_{13}$, facilitating the study of synergistic effects by which combinations of metals result in enhanced catalytic properties. Ultimately, the incorporation of oxide systems, specifically zirconium oxide ($Zr_{16}O_{32}$) and its doped variants ($RhZr_{16}O_{31}$, $LaZr_{15}O_{31}$), introduces a distinct category of materials that can function as active catalyst supports or direct catalytic centers, particularly for reactions involving oxygenated species.

The predominant implication of this dataset lies in its extensive scale, making it highly suitable for training advanced machine learning models. The extensive collection of over $425,000$ high-fidelity data points serves as an optimal foundation for the development of robust deep learning architectures, including graph neural networks, which are adept at discerning the intricate quantum-mechanical relationships among the structure, composition, and a catalyst's adsorbate interaction energies. Such models are capable of providing prompt and precise predictions of catalytic properties for novel, yet-to-be-synthesized nanoclusters, thus significantly hastening the screening and discovery processes for next-generation catalysts.

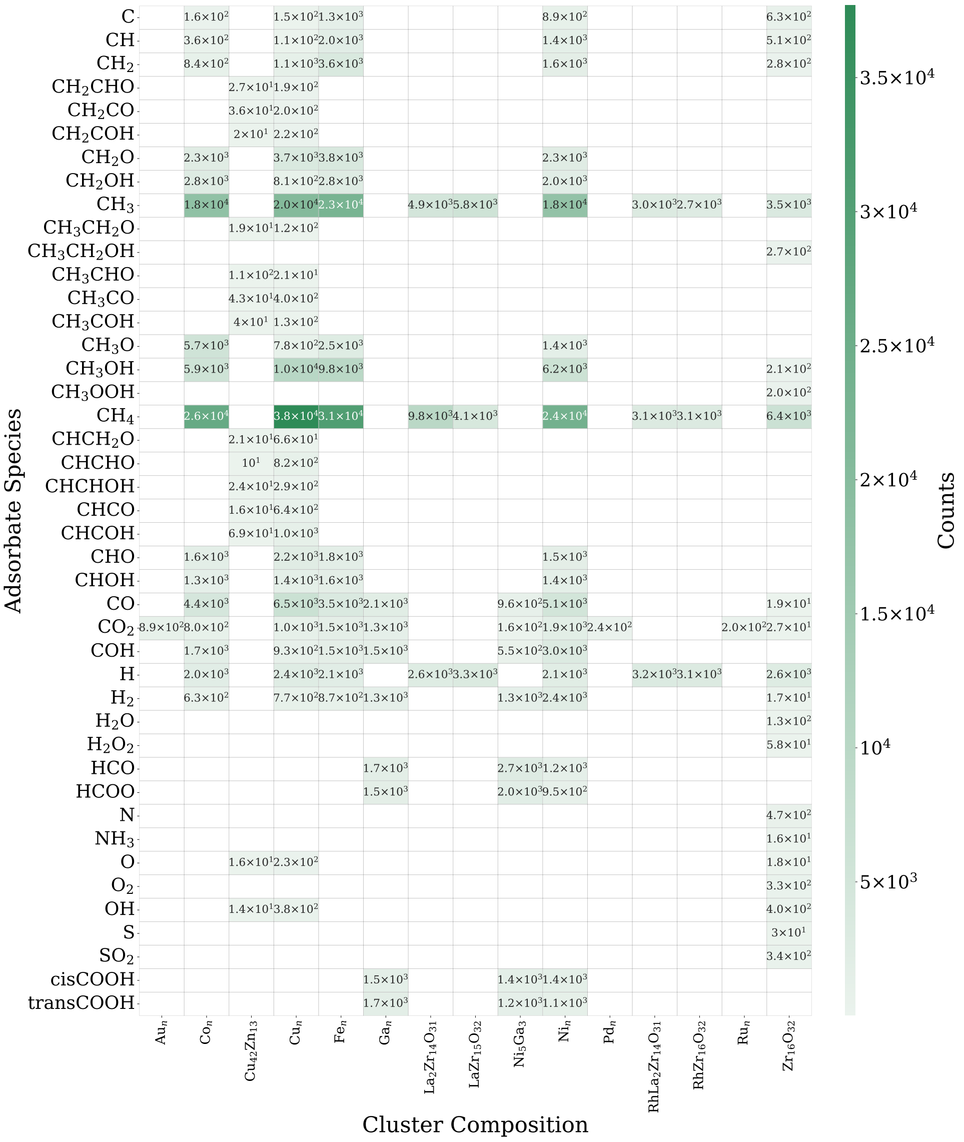

Figure S-1. Data set counts for combinations of nanocluster compositions and adsorbate species.

Figure S-1 illustrates a visual depiction of the quantity of various adsorbed systems within the dataset. It is evident that there is a substantial disparity in sample representativity, irrespective of the nanocluster's size, composition, or adsorbate, with a minority of each system type constituting the majority. The diversity in combinations of nanocluster composition and adsorbate is considerable, yet uneven, with numerous adsorbate species occurring in only a limited number of nanocluster compositions or being significantly underrepresented.

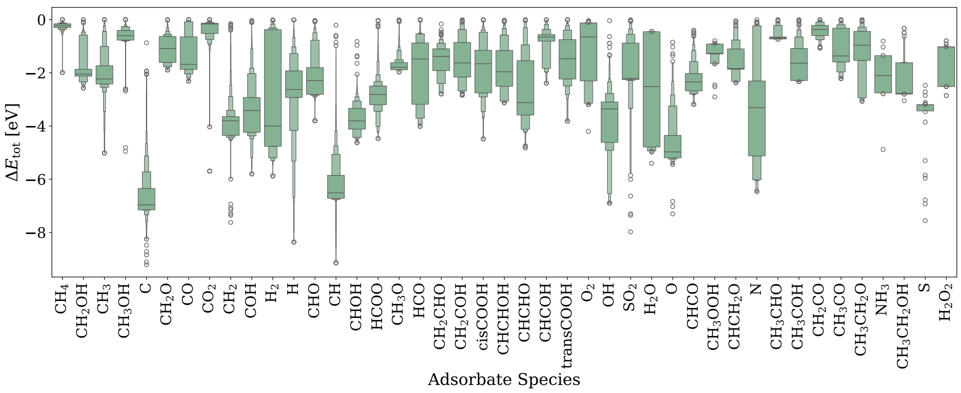

Figure S-2. Letter-value plots showing ΔE_{tot} distribution for adsorbate species in data set.

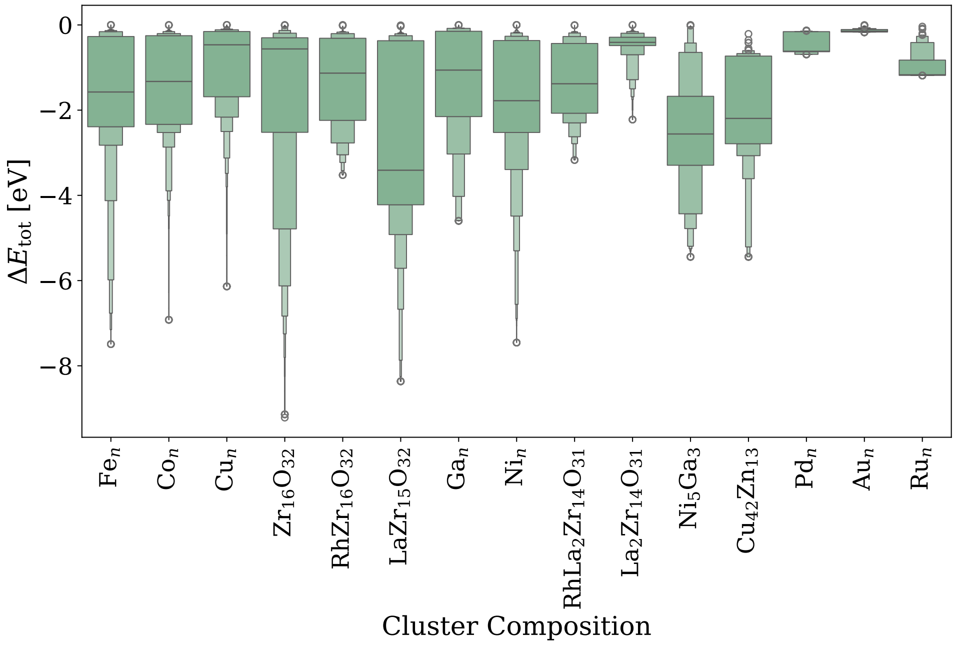

Figure S-3. Letter-value plots showing ΔE_{tot} distribution for nanocluster compositions in data set.

The distribution of the property of interest (ΔE_{tot}) across various adsorbates and nanocluster compositions is depicted in Figures S-2 and S-3. Analysis of the letter-value plots reveals that the property distribution exhibits significant variability among different adsorbate species, while it remains relatively uniform across most nanocluster compositions. For nanocluster compositions such as Pd_n, Au_n, and Ru_n, the property distribution is notably confined and concentrated within a narrow range. This phenomenon can be attributed to the limited diversity of adsorbates associated with these specific nanocluster compositions in the dataset. Conversely, for adsorbate

species, the diversity in potential interactions, including variations in orientations, nanocluster compositions, and adsorption sites, expands the distribution of property values, resulting in a greater incidence of outliers.

S-3 ADDITIONAL INFORMATION ON DESCRIPTOR OPTIMIZATION

Besides the analysis using the mean absolute error (MAE) presented in the main manuscript, here we also present the results on the structural descriptor optimization using the mean squared error (MSE) metric. Figure S-4 presents the MSE for the CM site size optimization and Figures S-5 and S-6 the MSE for (L)MBTR.

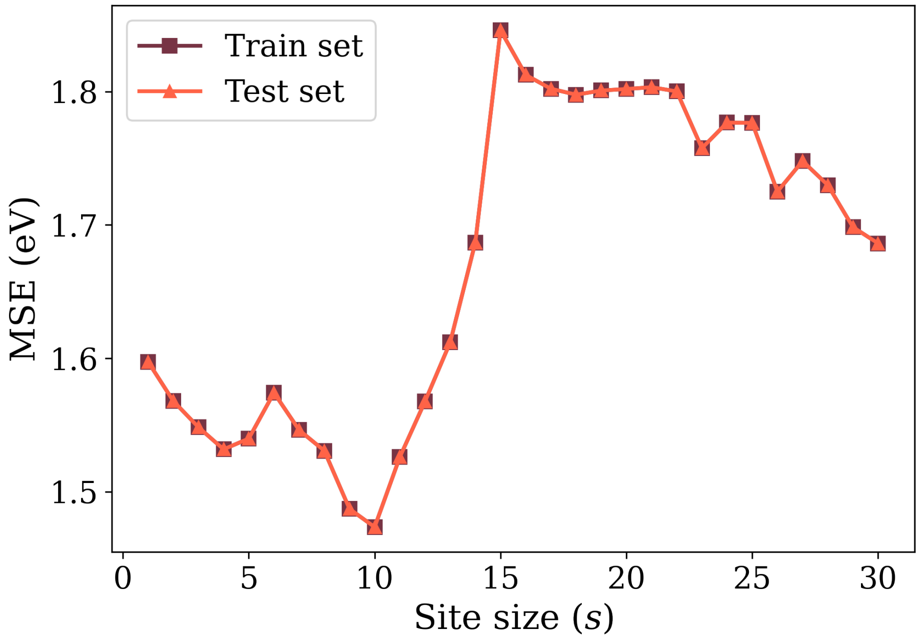

Figure S-4. Mean squared errors for CM linear optimization of the s parameter.

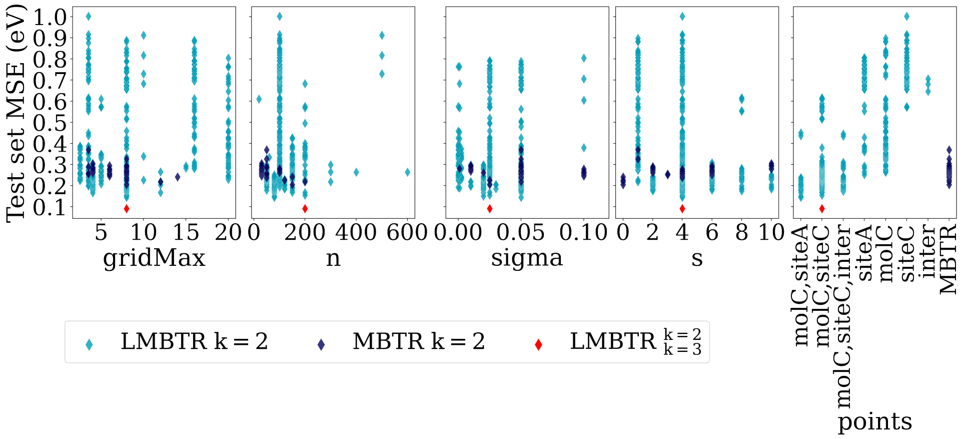

Figure S-5. Mean squared errors for MBTR $k = 2$ term parameters linear optimization.

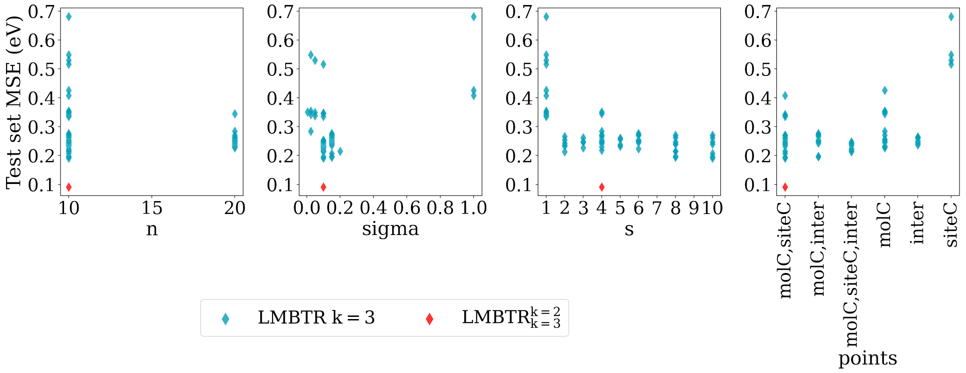

Figure S-6. Mean squared errors for MBTR $k = 3$ term parameters linear optimization.

S-4 RANDOM FOREST REGRESSOR DETAILED RESULTS

The `RandomForestRegressor` (RFR) algorithm from the Scikit-learn package was selected as the machine learning model to evaluate the best featurization protocols achieved. The following sections are separated by descriptor and detail results from the RFR model.

S-4.1 Coulomb Matrix in Data Set

Figure S-7 presents the prediction distribution for RFR with the CM eigenspectrum with $s = 9$. The MAE obtained for the train set is 0.019 eV and for the test set 0.048 eV. From Figure S-8 we see that there are few outliers with high prediction errors (>2 eV). High errors are associated with specific adsorbed systems, as observed in the highlighted cells in Figure S-9.

Notably, the $Zr_{16}O_{32}$ and $Cu_{42}Zn_{13}$ nanoclusters presented the largest errors, ranging from around 1.0 to 2.0 eV. In Figure S-1, we see that these systems are critically underrepresented for adsorbates with high errors, with only tens of examples for each, such as late CO_2 electrochemical reduction molecules that are limited to Cu_{55} and $Cu_{42}Zn_{13}$ nanoclusters and few exceptions. Nanoclusters of Cu composition also showed slightly larger errors around 1 to 0.4 eV for some adsorbates, which are also underrepresented. The oxygen atom showed the largest errors for all combinations because it is also scarce in the data set. The heavily represented nanoclusters can be identified by low MAEs ranging from 0.01 to 0.25 eV, with few exceptions such as Co-C, showing that there are other factors than representativity that affect the ability of the model.

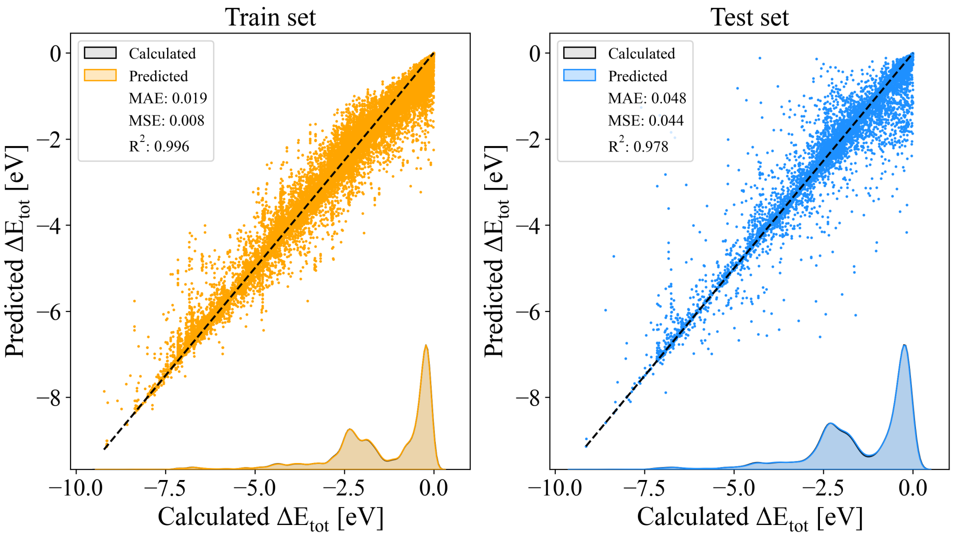

Figure S-7. RFR predictions for the CM with 9 site atoms.

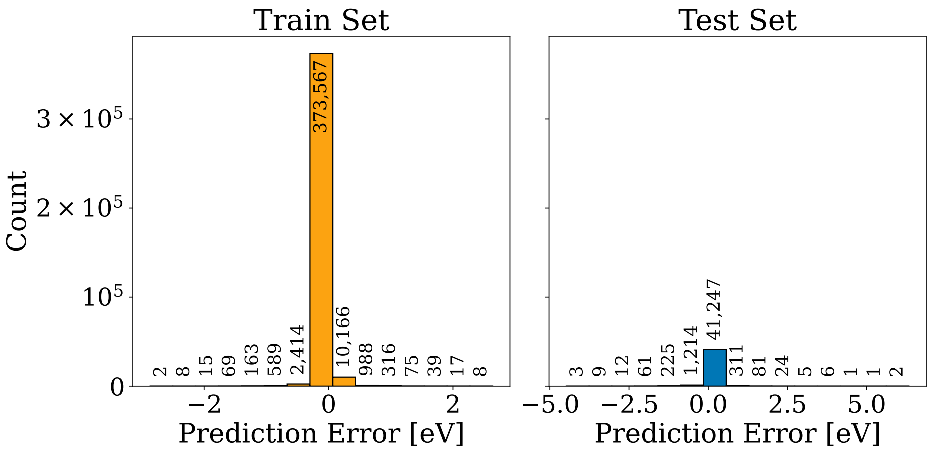

Figure S-8. CM with 9 site atoms with RFR model error histogram for train and test sets.

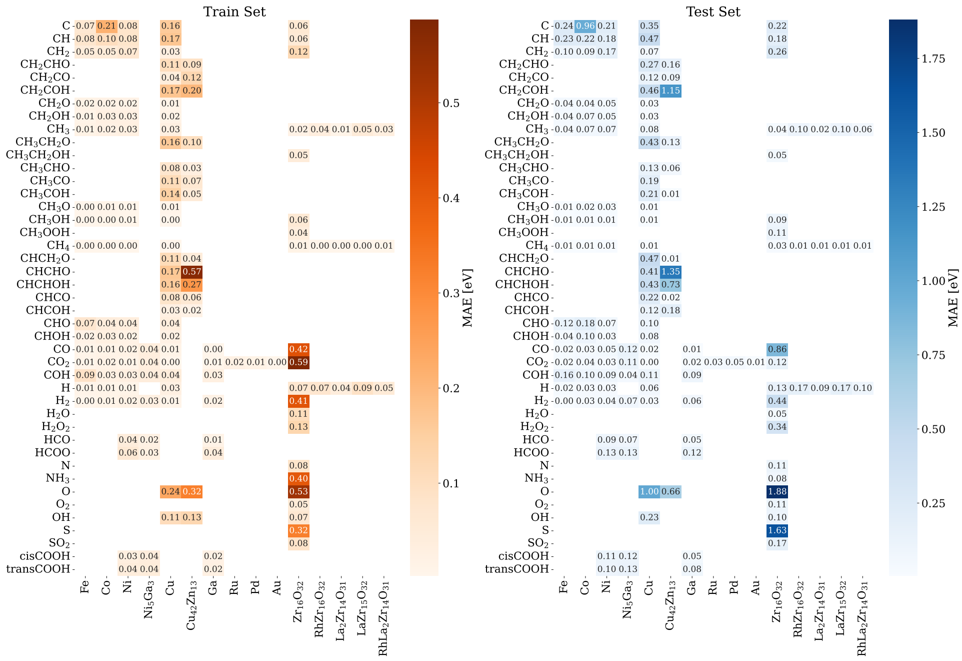

Figure S-9. CM with 9 site atoms with RFR model MAEs for different adsorbed systems.

S-4.2 LMBTR in Data Set

Due to a great number of features and the complexity of RFR, a principal component analysis (PCA) linear dimensionality reduction method[10] was utilized to reduce the total number of features of concatenated LMBTR. The two different descriptors ($k = 2$, $k = 3$) were independently scaled by the built-in DScribe normalization $L2$[11] and the resulting vector from concatenation was scaled using `StandardScaler` from Scikit-learn before performing the PCA transformation to ensure that both vectors are on the same

scale. Figures S-10 and S-11 present the prediction distribution for the PCA transformed LMBTR with the RFR algorithm. Figure S-12 details the mean absolute errors for different adsorbed systems.

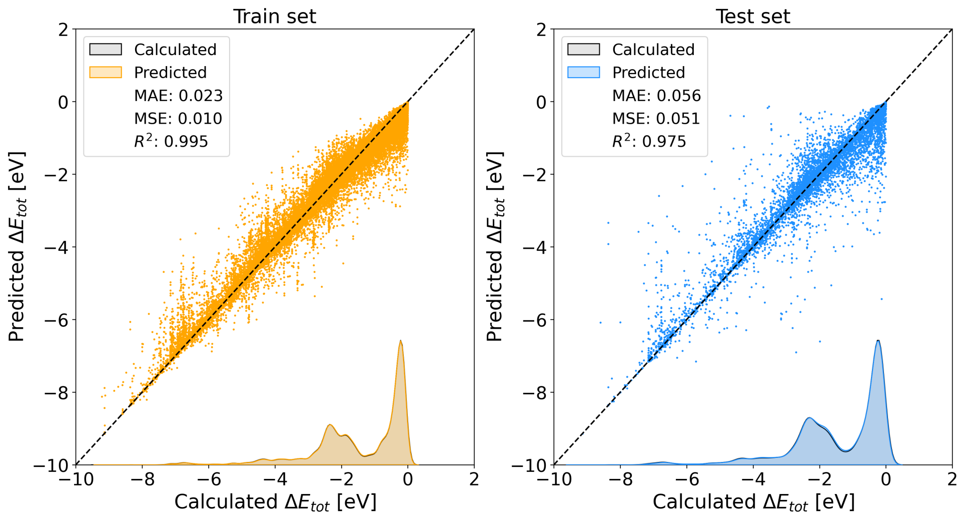

Figure S-10. RFR predictions for the PCA transformed concatenated LMBTR.

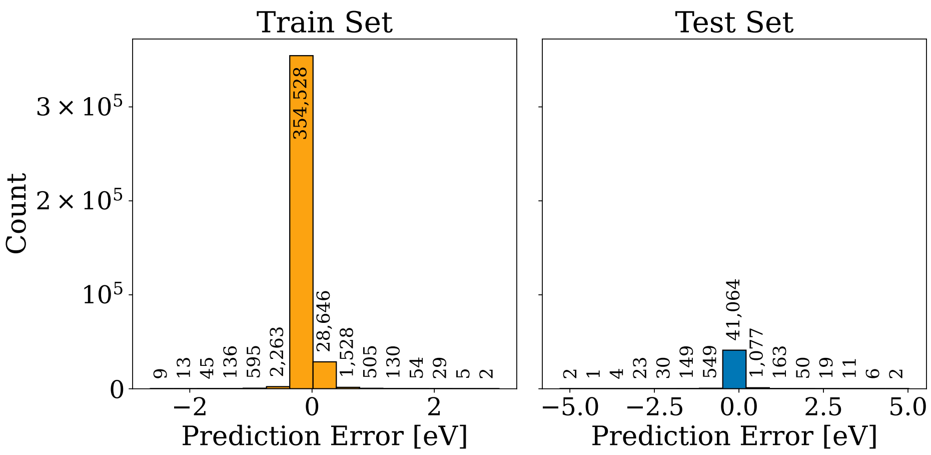

Figure S-11. PCA transformed concatenated LMBTR with RFR model error histogram for train and test sets.

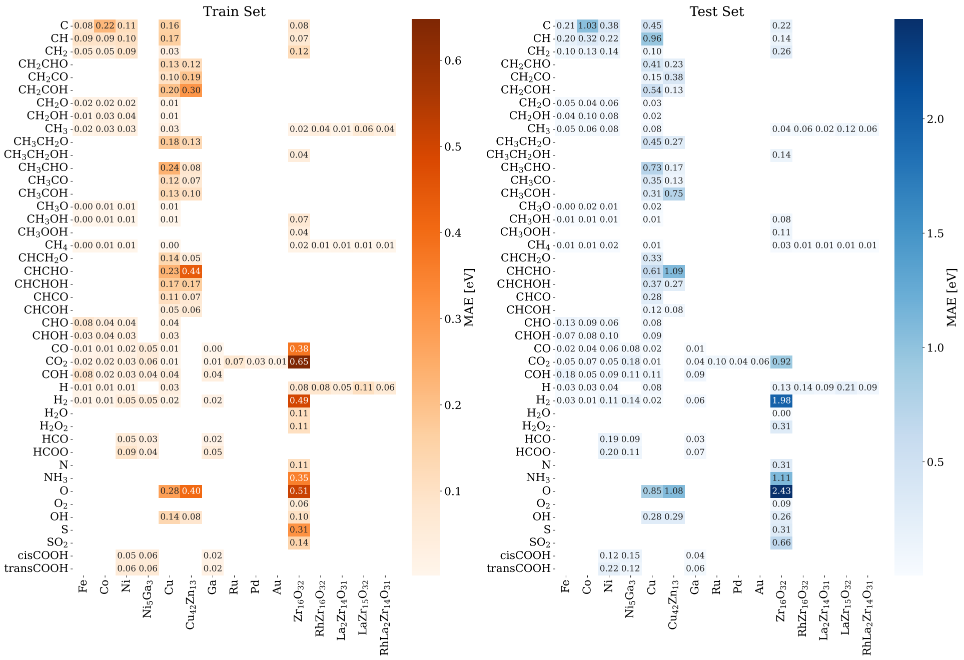

Figure S-12. PCA transformed concatenated LMBTR with the RFR model MAEs for combinations of nanocluster compositions and adsorbate species.

S-4.3 External Test Set Detailed Results

Figure S-13 presents the prediction distribution for the CM, LMBTR and CM with unpaired electrons with the RFR in the external test set.

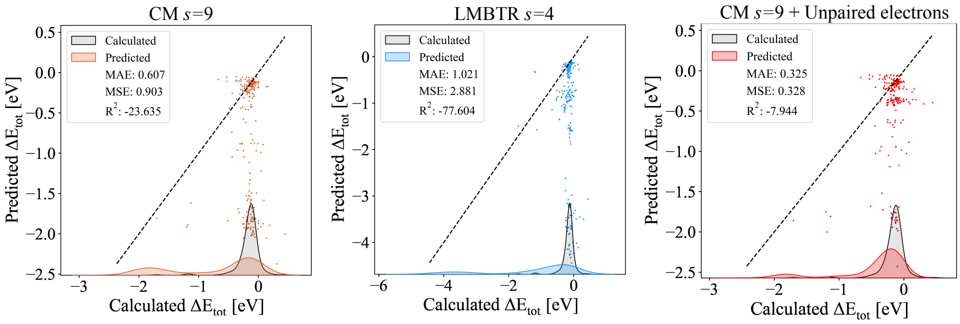

Figure S-13. Prediction distribution and errors for the RFR model which each descriptor on the external test set.

For assessing the possible influence of the loss of information from the PCA transformation in the LMBTR descriptor, a reconstruction error analysis was performed in the external test set with results presented in Table S-2. By comparing the reconstructed MSE per average variance in the external set it is possible to see that

molecules CH_3 and CH_4 (most occurences in data) have the smaller errors while the H_2O (not present in data set with Cu_{13} nanocluster) has the highest.

Table S-2. Scaled mean squared error (MSE), average variance (AvgVar), and MSE / AvgVar ratio for LMBTR PCA reconstruction in the external test set.

System	MSE (scaled)	AvgVar (scaled)	MSE / AvgVar
CH3 Cu13	0.003777	0.039436	0.095775
CH4 Cu13	0.002189	0.029704	0.073693
CH4O Cu13	0.004325	0.039938	0.108292
CO Cu13	0.009448	0.081755	0.115564
H2O Cu13	0.033690	0.078875	0.427131

S-5 TABLES

The following tables presents the test set MAE (testMAE) results for all conducted tests during the parameter optimization of MBTR and LMBTR with the ridge regression. The tables are provided separately for the MBTR and LMBTR $k = 2$ term (Tables S-3, S-4) and LMBTR $k = 3$ term (Table S-5. The provided parameters are the site size (siteSize), LMBTR center initialization positions (points), geometry function for the many-body term (geometry), maximum grid value (gridMax), grid resolution (n), kernel width (sigma) and exponential weighting scale (scale).

S-5.1 MBTR k=2 Optimization

Table S-3. Results for the optimization of the parameters of the MBTR with $k = 2$ descriptor using the linear regression.

testMAE	points	siteSize	geometry	gridMax	n	sigma	scale
0.261	MBTR	4.0	distance	8.0	50	0.05	0.0
0.262	MBTR	0.0	distance	8.0	150	0.025	0.2
0.265	MBTR	4.0	distance	6.0	30	0.1	0.2
0.269	MBTR	4.0	distance	3.5	50	0.05	0.0
0.27	MBTR	3.0	distance	3.5	50	0.05	0.0
0.271	MBTR	2.0	distance	4.0	100	0.02	0.2
0.272	MBTR	3.0	distance	8.0	50	0.05	0.0
0.273	MBTR	4.0	distance	4.0	30	0.1	0.2
0.275	MBTR	4.0	distance	6.0	30	0.05	0.2
0.276	MBTR	6.0	distance	6.0	30	0.1	0.2
0.277	MBTR	0.0	distance	8.0	120	0.025	0.5

Continued on next page

Table S-3 – continued from previous page

testMAE	points	siteSize	geometry	gridMax	n	sigma	scale
0.278	MBTR	0.0	distance	12.0	200	0.05	0.2
0.278	MBTR	4.0	distance	8.0	30	0.1	0.2
0.281	MBTR	2.0	distance	4.0	100	0.05	0.2
0.282	MBTR	6.0	distance	4.0	30	0.1	0.2
0.284	MBTR	6.0	distance	4.0	30	0.05	0.2
0.284	MBTR	6.0	distance	6.0	30	0.05	0.2
0.287	MBTR	6.0	distance	8.0	30	0.1	0.2
0.289	MBTR	2.0	distance	4.0	100	0.001	0.2
0.289	MBTR	4.0	distance	8.0	30	0.05	0.2
0.29	MBTR	2.0	distance	3.5	50	0.05	0.0
0.294	MBTR	2.0	distance	8.0	50	0.05	0.0
0.294	MBTR	0.0	distance	14.0	150	0.05	0.4
0.295	MBTR	4.0	distance	6.0	30	0.01	0.2
0.296	MBTR	4.0	distance	4.0	30	0.01	0.2
0.3	MBTR	6.0	distance	8.0	30	0.05	0.2
0.305	MBTR	6.0	distance	6.0	30	0.01	0.2
0.308	MBTR	6.0	distance	4.0	30	0.01	0.2
0.311	MBTR	1.0	distance	8.0	50	0.05	0.0
0.319	MBTR	10.0	distance	6.0	30	0.1	0.2
0.32	MBTR	6.0	distance	8.0	30	0.01	0.2
0.327	MBTR	1.0	distance	3.5	50	0.05	0.0
0.331	MBTR	10.0	distance	4.0	30	0.05	0.2
0.332	MBTR	10.0	distance	8.0	30	0.05	0.2
0.341	MBTR	10.0	distance	6.0	30	0.01	0.2

S-5.2 LMBTR k=2 Optimization

Table S-4. Results for the optimization of the parameters of the LMBTR with $k = 2$ descriptor using the linear regression.

testMAE	points	siteSize	geometry	gridMax	n	sigma	scale
0.21	molC,siteA	4.0	distance	8.0	80	0.05	0.2
0.213	molC,siteA	4.0	distance	8.0	80	0.02	0.2
0.215	molC,siteC	4.0	distance	8.0	200	0.025	0.2
0.22	molC,siteA	4.0	distance	4.0	80	0.02	0.2
0.228	molC,siteC	4.0	distance	8.0	150	0.025	0.3
0.228	molC,siteA	4.0	distance	4.0	80	0.05	0.2
0.231	molC,siteC	4.0	distance	12.0	200	0.05	0.2
0.232	molC,siteC,inter	4.0	distance	4.0	80	0.02	0.2
0.235	molC,siteA	4.0	distance	3.5	100	0.05	0.5
0.235	molC,siteA	4.0	distance	3.5	100	0.05	0.25
0.235	molC,siteC	1.0	distance	8.0	300	0.01	1.0
0.235	molC,siteA	4.0	distance	3.5	100	0.05	0.25
0.236	molC,siteC,inter	6.0	distance	4.0	80	0.02	0.2
0.237	molC,siteC	4.0	distance	8.0	150	0.05	0.3

Continued on next page

Table S-4 – continued from previous page

testMAE	points	siteSize	geometry	gridMax	n	sigma	scale
0.237	molC,siteC,inter	2.0	distance	8.0	80	0.05	0.2
0.238	molC,siteC,inter	4.0	distance	8.0	80	0.05	0.2
0.238	molC,siteC,inter	8.0	distance	4.0	80	0.02	0.2
0.238	molC,siteA	2.0	distance	8.0	80	0.05	0.2
0.239	molC,siteA	2.0	distance	4.0	80	0.02	0.2
0.24	molC,siteC,inter	2.0	distance	4.0	80	0.02	0.2
0.241	molC,siteA	4.0	distance	3.5	100	0.05	1.0
0.241	molC,siteC,inter	4.0	distance	4.0	80	0.05	0.2
0.241	molC,siteC,inter	6.0	distance	8.0	80	0.05	0.2
0.242	molC,siteC,inter	4.0	distance	8.0	80	0.02	0.2
0.242	molC,siteC,inter	8.0	distance	8.0	80	0.05	0.2
0.242	molC,siteA	2.0	distance	8.0	80	0.02	0.2
0.244	molC,siteA	2.0	distance	4.0	80	0.05	0.2
0.244	molC,siteC,inter	10.0	distance	4.0	80	0.02	0.2
0.244	molC,siteC,inter	2.0	distance	8.0	80	0.02	0.2
0.245	molC,siteC	4.0	distance	4.0	120	0.01	0.3
0.245	molC,siteC	4.0	distance	8.0	120	0.03	0.3
0.245	molC,siteA	4.0	distance	8.0	50	0.05	0.2
0.245	molC,siteC	4.0	distance	4.0	120	0.03	0.3
0.245	molC,siteC,inter	6.0	distance	4.0	80	0.05	0.2
0.246	molC,siteC,inter	6.0	distance	8.0	80	0.02	0.2
0.246	molC,siteC,inter	8.0	distance	4.0	80	0.05	0.2
0.246	molC,siteC,inter	8.0	distance	8.0	80	0.02	0.2
0.246	molC,siteC	4.0	distance	4.0	100	0.02	0.2
0.246	molC,siteC	4.0	distance	8.0	120	0.05	0.3
0.247	molC,siteC,inter	2.0	distance	4.0	80	0.05	0.2
0.248	molC,siteC,inter	10.0	distance	8.0	80	0.05	0.2
0.248	molC,siteA	4.0	distance	4.0	50	0.05	0.2
0.25	molC,siteC	6.0	distance	4.0	100	0.02	0.2
0.251	molC,siteC	2.0	distance	4.0	100	0.02	0.2
0.252	molC,siteC	4.0	distance	2.5	200	0.001	0.0
0.252	molC,siteC,inter	10.0	distance	4.0	80	0.05	0.2
0.253	molC,siteC,inter	10.0	distance	8.0	80	0.02	0.2
0.253	molC,siteC	4.0	distance	8.0	150	0.001	0.3
0.254	molC,siteC,inter	1.0	distance	10.0	150	0.001	1.0
0.254	molC,siteC	4.0	distance	3.5	100	0.025	0.25
0.255	molC,siteC	4.0	distance	3.5	100	0.025	0.5
0.255	molC,siteA	4.0	distance	8.0	50	0.02	0.2
0.255	molC,siteA	4.0	distance	4.0	50	0.02	0.2
0.255	molC,siteC	4.0	distance	8.0	100	0.05	0.2
0.256	molC,siteC	6.0	distance	8.0	100	0.05	0.2
0.256	molC,siteC	4.0	distance	2.5	200	0.0001	0.0
0.257	molC,siteC	2.0	distance	8.0	100	0.05	0.2
0.257	molC,siteC	4.0	distance	8.0	150	0.0001	0.3
0.258	molC,siteC	4.0	distance	8.0	120	0.01	0.3

Continued on next page

Table S-4 – continued from previous page

testMAE	points	siteSize	geometry	gridMax	n	sigma	scale
0.258	molC,siteC	4.0	distance	5.0	100	0.025	0.0
0.259	molC,siteC	8.0	distance	3.5	100	0.025	0.25
0.26	molC,siteC	4.0	distance	2.5	150	0.001	0.3
0.261	molC,siteC	1.0	distance	3.5	100	0.025	0.25
0.261	molC,siteC	4.0	distance	4.0	80	0.02	0.2
0.262	molC,siteC	1.0	distance	3.5	100	0.025	0.5
0.262	molC,siteC,inter	4.0	distance	4.0	50	0.05	0.2
0.263	molC,siteC	4.0	distance	3.5	100	0.025	1.0
0.264	molC,siteC,inter	2.0	distance	4.0	50	0.05	0.2
0.264	molC,siteA	2.0	distance	4.0	50	0.05	0.2
0.265	molC,siteC	4.0	distance	2.5	150	0.0001	0.3
0.265	molC,siteC	6.0	distance	8.0	100	0.02	0.2
0.265	molC,siteC	2.0	distance	4.0	80	0.02	0.2
0.265	molC,siteC,inter	6.0	distance	4.0	50	0.05	0.2
0.265	molC,siteC	4.0	distance	8.0	100	0.02	0.2
0.265	molC,siteC	6.0	distance	4.0	80	0.02	0.2
0.265	molC,siteC	4.0	distance	12.0	120	0.03	0.3
0.266	molC	4.0	distance	5.0	400	0.025	0.5
0.266	molC	4.0	distance	5.0	300	0.025	0.5
0.266	molC	4.0	distance	5.0	600	0.025	0.5
0.267	molC,siteC,inter	8.0	distance	4.0	50	0.05	0.2
0.267	molC	4.0	distance	5.0	200	0.025	0.5
0.267	molC,siteC	2.0	distance	8.0	100	0.02	0.2
0.268	molC,siteC	4.0	distance	4.0	120	0.05	0.3
0.269	molC,siteC	4.0	distance	4.0	100	0.05	0.2
0.269	molC,siteC	4.0	distance	2.5	200	0.025	0.0
0.269	molC,siteC	4.0	distance	2.5	150	0.025	0.3
0.27	molC,siteC	4.0	distance	2.5	100	0.025	0.0
0.27	molC,siteC	8.0	distance	4.0	80	0.02	0.2
0.271	molC,siteC	4.0	distance	8.0	80	0.05	0.2
0.271	molC,siteC	1.0	distance	3.5	100	0.025	1.0
0.271	molC,siteC,inter	4.0	distance	4.0	50	0.02	0.2
0.271	molC,siteC	4.0	distance	4.0	80	0.05	0.2
0.271	molC,siteC	2.0	distance	4.0	100	0.05	0.2
0.271	molC,siteC	8.0	distance	3.5	100	0.025	1.0
0.272	molC,siteC	2.0	distance	4.0	80	0.05	0.2
0.272	molC,siteC	6.0	distance	4.0	100	0.05	0.2
0.272	molC,siteC	4.0	distance	5.0	100	0.001	0.0
0.272	molC,siteA	2.0	distance	4.0	50	0.02	0.2
0.273	molC,siteC	2.0	distance	8.0	80	0.05	0.2
0.273	molC,siteC	4.0	distance	8.0	80	0.02	0.2
0.273	molC	4.0	distance	5.0	150	0.025	0.5
0.273	molC,siteC	4.0	inverse	5.0	150	0.0025	0.0
0.274	molC,siteC	4.0	distance	12.0	120	0.01	0.3
0.274	molC,siteC,inter	2.0	distance	8.0	50	0.05	0.2

Continued on next page

Table S-4 – continued from previous page

testMAE	points	siteSize	geometry	gridMax	n	sigma	scale
0.274	molC,siteC	1.0	distance	3.5	100	0.05	0.25
0.274	molC,siteC,inter	10.0	distance	4.0	50	0.05	0.2
0.274	molC,siteC,inter	4.0	distance	8.0	50	0.05	0.2
0.274	molC,siteC	6.0	distance	4.0	80	0.05	0.2
0.274	molC,siteC	1.0	distance	3.5	100	0.05	0.25
0.274	molC,siteC	1.0	distance	3.5	100	0.05	0.25
0.274	molC,siteC	6.0	distance	8.0	80	0.05	0.2
0.275	molC,siteC	10.0	distance	4.0	80	0.02	0.2
0.275	molC,siteC	1.0	distance	3.5	100	0.05	0.5
0.275	molC,siteC	4.0	distance	5.0	100	0.0001	0.0
0.276	molC,siteC,inter	8.0	distance	8.0	50	0.05	0.2
0.276	molC,siteA	2.0	distance	8.0	50	0.05	0.2
0.277	molC,siteC	6.0	inverse	5.0	150	0.0025	0.0
0.277	molC,siteC	8.0	distance	4.0	100	0.05	0.2
0.277	molC,siteC	4.0	inverse	20.0	150	0.0025	0.0
0.277	molC,siteC	2.0	distance	8.0	80	0.02	0.2
0.277	molC,siteC	1.0	distance	8.0	100	0.05	0.25
0.277	molC,siteC	1.0	distance	8.0	100	0.05	0.25
0.277	molC,siteC	1.0	distance	8.0	100	0.05	0.25
0.278	molC,siteC,inter	2.0	distance	4.0	50	0.02	0.2
0.279	molC,siteC,inter	8.0	distance	4.0	50	0.02	0.2
0.279	molC,siteC	4.0	distance	2.5	100	0.001	0.0
0.279	molC,siteC,inter	6.0	distance	8.0	50	0.05	0.2
0.279	molC,siteC	6.0	distance	8.0	80	0.02	0.2
0.279	molC,siteC	8.0	distance	8.0	80	0.05	0.2
0.279	molC,siteC	8.0	distance	4.0	80	0.05	0.2
0.28	molC,siteC	4.0	distance	3.5	100	0.05	0.25
0.28	molC,siteC	1.0	distance	8.0	100	0.05	0.5
0.28	molC,siteC	1.0	distance	8.0	100	0.05	0.5
0.28	molC,siteC,inter	6.0	distance	4.0	50	0.02	0.2
0.281	molC,siteC	8.0	distance	8.0	80	0.02	0.2
0.281	molC	1.0	distance	8.0	300	0.01	1.0
0.281	molC,siteC,inter	4.0	distance	8.0	50	0.02	0.2
0.281	molC,siteC	4.0	inverse	20.0	150	0.001	0.0
0.281	molC,siteC	4.0	distance	3.5	100	0.05	0.5
0.283	molC,siteC	4.0	inverse	5.0	150	0.0001	0.0
0.283	molC,siteC	6.0	inverse	20.0	150	0.0025	0.0
0.283	molC,siteC,inter	2.0	distance	8.0	50	0.02	0.2
0.284	molC,siteC	4.0	inverse	5.0	150	1e-05	0.0
0.284	molC,siteC,inter	8.0	distance	8.0	50	0.02	0.2
0.284	molC,siteA	2.0	distance	8.0	50	0.02	0.2
0.285	molC,siteC	4.0	inverse	20.0	150	0.0001	0.0
0.285	molC,siteC	4.0	inverse	20.0	150	0.0001	0.0
0.285	molC,siteC	4.0	distance	2.5	100	0.0001	0.0
0.286	molC,siteC	10.0	distance	8.0	80	0.05	0.2

Continued on next page

Table S-4 – continued from previous page

testMAE	points	siteSize	geometry	gridMax	n	sigma	scale
0.286	molC,siteC	6.0	inverse	5.0	150	0.0001	0.0
0.286	molC,siteC	4.0	inverse	20.0	150	1e-05	0.0
0.287	molC,siteC	10.0	distance	4.0	80	0.05	0.2
0.287	molC,siteC	4.0	distance	3.5	100	0.05	1.0
0.287	molC,siteC,inter	10.0	distance	8.0	50	0.05	0.2
0.287	molC,siteC,inter	6.0	distance	8.0	50	0.02	0.2
0.287	molC,siteC	6.0	inverse	5.0	150	1e-05	0.0
0.287	molC,siteC,inter	10.0	distance	4.0	50	0.02	0.2
0.288	molC,siteC	10.0	distance	8.0	80	0.02	0.2
0.288	molC,siteC	4.0	distance	8.0	100	0.05	0.25
0.289	molC	1.0	distance	5.0	100	0.025	0.4
0.289	molC	1.0	distance	5.0	100	0.025	0.3
0.289	molC,siteC	6.0	inverse	20.0	150	0.001	0.0
0.29	molC,siteC	8.0	distance	8.0	100	0.025	0.25
0.29	molC	1.0	distance	5.0	100	0.025	0.5
0.29	molC	4.0	distance	5.0	100	0.025	0.5
0.291	molC	4.0	distance	5.0	150	0.025	0.0
0.291	molC,siteC	8.0	distance	3.5	100	0.05	0.25
0.293	molC,siteC	6.0	inverse	20.0	150	0.0001	0.0
0.293	molC,siteC	6.0	inverse	20.0	150	0.0001	0.0
0.294	molC,siteC	6.0	inverse	20.0	150	1e-05	0.0
0.294	siteA	4.0	distance	8.0	100	0.025	0.1
0.294	molC,siteC	1.0	distance	8.0	100	0.025	0.5
0.295	molC,siteC	8.0	distance	8.0	100	0.025	0.5
0.295	molC,siteC	4.0	distance	8.0	100	0.025	0.25
0.296	molC,siteC	4.0	distance	4.0	50	0.05	0.2
0.296	molC	4.0	distance	2.5	150	0.025	0.0
0.296	molC,siteC,inter	10.0	distance	8.0	50	0.02	0.2
0.296	molC,siteC,inter	4.0	distance	16.0	100	0.05	0.25
0.297	molC,siteC,inter	4.0	distance	16.0	100	0.05	0.5
0.297	molC,siteC	2.0	distance	4.0	50	0.05	0.2
0.297	molC	4.0	distance	2.5	150	0.001	0.0
0.298	molC,siteC	4.0	distance	8.0	100	0.025	0.5
0.299	molC,siteC	1.0	distance	8.0	100	0.05	1.0
0.299	molC,siteC	1.0	distance	8.0	100	0.05	1.0
0.299	molC,siteC	4.0	distance	20.0	150	0.05	0.3
0.3	molC	1.0	distance	3.5	100	0.025	0.25
0.3	molC,siteC	4.0	distance	8.0	100	0.05	1.0
0.3	molC,siteC	6.0	distance	4.0	50	0.05	0.2
0.301	molC,siteC	4.0	inverse	2.5	150	0.0025	0.0
0.302	molC	4.0	distance	5.0	150	0.001	0.0
0.302	molC,siteC,inter	4.0	distance	16.0	100	0.025	0.25
0.303	molC	4.0	distance	2.5	150	0.0001	0.0
0.303	molC,siteC	6.0	inverse	2.5	150	0.0025	0.0
0.303	molC,siteC,inter	4.0	distance	16.0	100	0.025	0.5

Continued on next page

Table S-4 – continued from previous page

testMAE	points	siteSize	geometry	gridMax	n	sigma	scale
0.304	molC	1.0	distance	3.5	100	0.025	0.5
0.304	molC	4.0	distance	5.0	150	0.0001	0.0
0.305	siteA	4.0	distance	12.0	100	0.05	0.1
0.306	molC,siteC	4.0	inverse	20.0	150	0.025	0.0
0.306	molC,siteC	8.0	distance	4.0	50	0.05	0.2
0.307	molC,siteC	4.0	distance	2.5	150	0.05	0.3
0.307	molC,siteC	4.0	distance	2.5	200	0.05	0.0
0.308	molC,siteC,inter	4.0	distance	16.0	100	0.05	1.0
0.308	molC,siteC	4.0	distance	2.5	100	0.05	0.0
0.309	molC,siteC	4.0	distance	4.0	50	0.02	0.2
0.309	molC,siteC	6.0	inverse	20.0	150	0.025	0.0
0.309	molC	1.0	distance	10.0	200	0.001	1.0
0.311	molC,siteC	6.0	inverse	20.0	150	0.025	0.0
0.311	molC,siteC	4.0	distance	8.0	50	0.05	0.2
0.311	molC,siteC	4.0	inverse	20.0	150	0.025	0.0
0.312	molC,siteC	6.0	distance	8.0	50	0.05	0.2
0.312	molC,siteC	4.0	inverse	20.0	150	0.001	0.0
0.312	molC,siteC	2.0	distance	4.0	50	0.02	0.2
0.313	molC,siteC	2.0	distance	8.0	50	0.05	0.2
0.313	molC,siteC	4.0	inverse	2.5	150	0.0001	0.0
0.313	molC,siteC	6.0	inverse	2.5	150	0.0001	0.0
0.314	molC,siteC	6.0	inverse	2.5	150	1e-05	0.0
0.314	molC,siteC	10.0	distance	4.0	50	0.05	0.2
0.314	molC,siteC	4.0	inverse	2.5	150	1e-05	0.0
0.315	molC,siteC,inter	4.0	distance	16.0	100	0.025	1.0
0.315	molC,siteC	4.0	distance	8.0	50	0.02	0.2
0.315	molC,siteC	1.0	distance	8.0	100	0.025	1.0
0.316	molC,siteC	8.0	distance	4.0	50	0.02	0.2
0.316	molC	1.0	distance	3.5	100	0.025	1.0
0.316	molC,siteC	6.0	distance	4.0	50	0.02	0.2
0.317	molC,siteC	8.0	distance	8.0	50	0.05	0.2
0.317	molC,siteC	4.0	inverse	20.0	150	0.0001	0.0
0.317	molC,siteC	2.0	distance	8.0	50	0.02	0.2
0.318	molC,siteC	4.0	distance	20.0	150	0.025	0.3
0.319	molC,siteC	6.0	distance	8.0	50	0.02	0.2
0.32	siteA	4.0	distance	15.0	100	0.05	0.1
0.321	molC	4.0	distance	5.0	60	0.025	0.5
0.322	molC,siteC	8.0	distance	8.0	50	0.02	0.2
0.324	molC	1.0	distance	3.5	100	0.05	0.25
0.324	molC,siteC	6.0	inverse	20.0	150	0.001	0.0
0.324	molC,siteC	10.0	distance	4.0	50	0.02	0.2
0.325	molC,siteC	10.0	distance	8.0	50	0.05	0.2
0.326	molC	4.0	inverse	2.5	200	0.0025	0.0
0.328	molC	1.0	distance	3.5	100	0.05	0.5
0.328	molC	4.0	inverse	5.0	200	0.0025	0.0

Continued on next page

Table S-4 – continued from previous page

testMAE	points	siteSize	geometry	gridMax	n	sigma	scale
0.329	siteA	4.0	distance	15.0	100	0.025	0.1
0.329	molC,siteC	6.0	inverse	20.0	150	0.0001	0.0
0.33	molC,siteC	10.0	distance	8.0	50	0.02	0.2
0.332	molC	4.0	inverse	5.0	150	0.0025	0.0
0.338	molC,siteC	4.0	distance	20.0	150	0.001	0.3
0.34	molC	1.0	distance	3.5	100	0.05	1.0
0.34	molC	4.0	inverse	20.0	200	0.0025	0.0
0.34	molC	4.0	inverse	2.5	200	1e-05	0.0
0.341	molC	4.0	inverse	20.0	200	0.001	0.0
0.341	molC	4.0	inverse	2.5	200	0.0001	0.0
0.343	molC	4.0	inverse	2.5	150	0.0025	0.0
0.343	molC	4.0	inverse	5.0	200	0.0001	0.0
0.344	molC	4.0	inverse	5.0	150	0.0001	0.0
0.344	molC	4.0	inverse	5.0	200	1e-05	0.0
0.344	molC	4.0	inverse	5.0	150	1e-05	0.0
0.344	molC	1.0	distance	8.0	100	0.05	0.25
0.345	molC	4.0	inverse	20.0	200	0.0001	0.0
0.345	molC	4.0	inverse	20.0	200	0.0001	0.0
0.346	molC	4.0	inverse	20.0	200	1e-05	0.0
0.351	molC	1.0	distance	8.0	100	0.05	0.5
0.352	molC	4.0	inverse	20.0	150	0.0025	0.0
0.352	molC,siteC	4.0	distance	20.0	100	0.025	0.0
0.358	molC	4.0	inverse	20.0	150	0.0001	0.0
0.358	molC	4.0	inverse	2.5	150	1e-05	0.0
0.358	molC	4.0	inverse	2.5	150	0.0001	0.0
0.358	molC	4.0	inverse	20.0	150	1e-05	0.0
0.359	molC	1.0	distance	8.0	100	0.025	0.25
0.362	molC	4.0	inverse	20.0	200	0.001	0.0
0.364	siteA	4.0	distance	16.0	100	0.05	0.25
0.365	molC	4.0	inverse	20.0	200	0.0001	0.0
0.365	siteA	4.0	distance	3.5	100	0.05	0.5
0.367	siteA	4.0	distance	3.5	100	0.05	0.25
0.368	molC	4.0	inverse	20.0	100	0.025	0.0
0.368	molC	1.0	distance	8.0	100	0.025	0.5
0.369	molC	1.0	distance	8.0	100	0.05	1.0
0.37	siteA	4.0	distance	16.0	100	0.025	0.25
0.373	siteA	4.0	distance	16.0	100	0.025	0.5
0.373	molC,siteC	4.0	distance	2.5	200	0.1	0.0
0.375	molC	4.0	inverse	20.0	200	0.025	0.0
0.378	molC	4.0	inverse	20.0	100	0.001	0.0
0.38	molC	4.0	inverse	20.0	100	0.0001	0.0
0.387	molC	4.0	distance	20.0	150	0.025	0.0
0.391	molC	1.0	distance	8.0	100	0.025	1.0
0.393	molC	4.0	inverse	20.0	200	0.025	0.0
0.394	molC,siteC,inter	4.0	distance	16.0	100	1.0	0.5

Continued on next page

Table S-4 – continued from previous page

testMAE	points	siteSize	geometry	gridMax	n	sigma	scale
0.396	molC	4.0	inverse	20.0	100	0.025	0.0
0.398	siteA	4.0	distance	16.0	100	0.025	1.0
0.403	molC,siteC,inter	4.0	distance	16.0	100	1.0	0.25
0.404	molC,siteA	4.0	distance	3.5	100	1.0	0.25
0.409	molC	4.0	inverse	20.0	100	0.001	0.0
0.411	molC	4.0	distance	20.0	150	0.001	0.0
0.411	molC	4.0	inverse	20.0	100	0.05	0.0
0.412	molC,siteA	4.0	distance	3.5	100	1.0	1.0
0.413	molC	4.0	distance	20.0	150	0.0001	0.0
0.413	molC	4.0	inverse	20.0	100	0.0001	0.0
0.416	molC	1.0	distance	16.0	100	0.05	0.25
0.425	molC	1.0	distance	16.0	100	0.05	0.5
0.427	molC	1.0	distance	16.0	100	0.025	0.25
0.437	molC	1.0	distance	16.0	100	0.025	0.5
0.444	molC	4.0	inverse	20.0	200	0.025	0.0
0.45	molC	1.0	distance	16.0	100	0.05	1.0
0.459	molC,siteC	4.0	distance	8.0	100	1.0	0.25
0.459	molC	1.0	distance	16.0	100	0.025	1.0
0.462	molC,siteC	4.0	distance	8.0	100	1.0	0.5
0.462	molC,siteC	4.0	distance	16.0	100	1.0	0.5
0.467	siteA	4.0	distance	3.5	100	1.0	0.5
0.472	molC,siteC	1.0	distance	8.0	100	1.0	1.0
0.477	molC	4.0	inverse	20.0	100	0.1	0.0
0.482	siteC	1.0	distance	5.0	100	0.025	0.4
0.482	siteC	1.0	distance	5.0	100	0.025	0.3
0.483	siteC	1.0	distance	5.0	100	0.025	0.5
0.484	siteA	4.0	distance	3.5	100	1.0	1.0
0.489	molC,siteC	4.0	distance	3.5	100	1.0	0.25
0.493	molC,siteC	1.0	distance	3.5	100	1.0	1.0
0.493	molC,siteC	4.0	distance	16.0	100	1.0	1.0
0.493	molC	4.0	inverse	20.0	200	0.001	0.0
0.494	molC,siteC	4.0	distance	8.0	100	1.0	1.0
0.495	molC,siteC	8.0	distance	16.0	100	1.0	0.5
0.496	molC,siteC	8.0	distance	8.0	100	1.0	0.5
0.498	molC	4.0	inverse	20.0	200	0.0001	0.0
0.507	inter	4.0	inverse	20.0	100	0.025	0.0
0.511	siteC	1.0	distance	8.0	100	0.05	0.25
0.511	siteC	1.0	distance	8.0	100	0.05	0.25
0.511	siteA	1.0	distance	8.0	100	0.05	0.25
0.511	siteA	1.0	distance	8.0	100	0.05	0.25
0.511	siteA	1.0	distance	8.0	100	0.05	0.25
0.511	siteC	1.0	distance	8.0	100	0.05	0.25
0.512	molC,siteC	4.0	distance	3.5	100	1.0	0.5
0.516	siteC	1.0	distance	8.0	100	0.025	0.25
0.516	siteC	1.0	distance	8.0	100	0.025	0.25

Continued on next page

Table S-4 – continued from previous page

testMAE	points	siteSize	geometry	gridMax	n	sigma	scale
0.516	siteA	1.0	distance	8.0	100	0.025	0.25
0.516	siteA	1.0	distance	8.0	100	0.025	0.25
0.516	siteA	1.0	distance	8.0	100	0.025	0.25
0.516	siteC	1.0	distance	8.0	100	0.025	0.25
0.517	siteC	1.0	distance	8.0	100	0.05	0.5
0.517	siteA	1.0	distance	8.0	100	0.05	0.5
0.517	siteA	1.0	distance	8.0	100	0.05	0.5
0.517	siteA	1.0	distance	8.0	100	0.05	0.5
0.517	siteC	1.0	distance	8.0	100	0.05	0.5
0.517	siteC	1.0	distance	8.0	100	0.05	0.5
0.517	inter	4.0	inverse	20.0	100	0.05	0.0
0.518	molC,siteC	4.0	distance	3.5	100	1.0	1.0
0.522	molC	4.0	distance	5.0	20	0.025	0.5
0.525	siteA	1.0	distance	8.0	100	0.025	0.5
0.525	siteA	1.0	distance	8.0	100	0.025	0.5
0.525	siteA	1.0	distance	8.0	100	0.025	0.5
0.525	siteC	1.0	distance	8.0	100	0.025	0.5
0.525	siteC	1.0	distance	8.0	100	0.025	0.5
0.525	siteC	1.0	distance	8.0	100	0.025	0.5
0.526	molC,siteC	8.0	distance	3.5	100	1.0	0.25
0.529	siteC	1.0	distance	10.0	200	0.001	1.0
0.531	inter	4.0	inverse	20.0	100	0.1	0.0
0.534	molC,siteC	8.0	distance	16.0	100	1.0	1.0
0.537	molC,siteC	8.0	distance	3.5	100	1.0	0.5
0.539	siteA	1.0	distance	16.0	100	0.05	0.25
0.539	siteC	1.0	distance	16.0	100	0.05	0.25
0.539	siteA	1.0	distance	16.0	100	0.05	0.25
0.539	siteA	1.0	distance	16.0	100	0.05	0.25
0.539	siteC	1.0	distance	16.0	100	0.05	0.25
0.539	siteC	1.0	distance	16.0	100	0.05	0.25
0.541	siteC	1.0	distance	16.0	100	1.0	0.5
0.541	siteA	1.0	distance	16.0	100	1.0	0.5
0.541	siteC	1.0	distance	16.0	100	1.0	0.5
0.541	siteA	1.0	distance	16.0	100	1.0	0.5
0.541	siteA	1.0	distance	16.0	100	1.0	0.5
0.541	siteC	1.0	distance	16.0	100	1.0	0.5
0.542	siteC	1.0	distance	8.0	100	1.0	0.5
0.542	siteC	1.0	distance	8.0	100	1.0	0.5
0.542	siteA	1.0	distance	8.0	100	1.0	0.5
0.542	siteA	1.0	distance	8.0	100	1.0	0.5
0.542	siteA	1.0	distance	8.0	100	1.0	0.5
0.542	siteC	1.0	distance	8.0	100	1.0	0.5
0.544	siteC	1.0	distance	3.5	100	0.025	0.25
0.544	siteC	1.0	distance	3.5	100	0.025	0.25
0.544	siteA	1.0	distance	3.5	100	0.025	0.25

Continued on next page

Table S-4 – continued from previous page

testMAE	points	siteSize	geometry	gridMax	n	sigma	scale
0.544	siteC	4.0	distance	8.0	100	0.05	0.25
0.544	siteC	1.0	distance	3.5	100	0.025	0.25
0.544	siteA	1.0	distance	3.5	100	0.025	0.25
0.544	siteA	1.0	distance	3.5	100	0.025	0.25
0.545	siteA	1.0	distance	16.0	100	0.05	0.5
0.545	siteC	1.0	distance	16.0	100	0.05	0.5
0.545	siteA	1.0	distance	16.0	100	0.05	0.5
0.545	siteC	1.0	distance	16.0	100	0.05	0.5
0.545	siteC	1.0	distance	16.0	100	0.05	0.5
0.547	siteC	1.0	distance	16.0	100	0.025	0.25
0.547	siteA	1.0	distance	16.0	100	0.025	0.25
0.547	siteC	1.0	distance	8.0	100	0.05	1.0
0.547	siteC	1.0	distance	16.0	100	0.025	0.25
0.547	siteC	1.0	distance	8.0	100	0.05	1.0
0.547	siteA	1.0	distance	16.0	100	0.025	0.25
0.547	siteA	1.0	distance	8.0	100	0.05	1.0
0.547	siteC	4.0	distance	8.0	100	0.025	0.25
0.547	siteA	1.0	distance	16.0	100	0.025	0.25
0.547	siteC	1.0	distance	16.0	100	0.025	0.25
0.547	siteA	1.0	distance	8.0	100	0.05	1.0
0.547	siteA	1.0	distance	8.0	100	0.05	1.0
0.547	siteC	1.0	distance	8.0	100	0.05	1.0
0.548	siteC	1.0	distance	3.5	100	0.05	0.25
0.548	siteA	1.0	distance	3.5	100	0.05	0.25
0.548	siteC	1.0	distance	3.5	100	0.05	0.25
0.548	siteC	1.0	distance	3.5	100	0.05	0.25
0.548	siteA	1.0	distance	3.5	100	0.05	0.25
0.548	siteA	1.0	distance	3.5	100	0.05	0.25
0.55	siteA	1.0	distance	3.5	100	0.025	0.5
0.55	siteC	1.0	distance	3.5	100	0.025	0.5
0.55	siteC	1.0	distance	3.5	100	0.025	0.5
0.55	siteA	1.0	distance	3.5	100	0.025	0.5
0.55	siteC	1.0	distance	3.5	100	0.025	0.5
0.55	siteA	1.0	distance	3.5	100	0.025	0.5
0.551	siteC	4.0	distance	8.0	100	0.05	0.5
0.551	siteC	4.0	inverse	20.0	100	0.025	0.0
0.552	siteC	1.0	distance	3.5	100	0.05	0.5
0.552	siteA	1.0	distance	3.5	100	0.05	0.5
0.552	siteC	1.0	distance	3.5	100	0.05	0.5
0.552	siteA	1.0	distance	3.5	100	0.05	0.5
0.552	siteC	1.0	distance	3.5	100	0.05	0.5
0.552	siteA	1.0	distance	3.5	100	0.05	0.5
0.553	siteA	1.0	distance	3.5	100	1.0	0.5
0.553	siteA	1.0	distance	3.5	100	1.0	0.5
0.553	siteA	1.0	distance	3.5	100	1.0	0.5

Continued on next page

Table S-4 – continued from previous page

testMAE	points	siteSize	geometry	gridMax	n	sigma	scale
0.553	siteC	1.0	distance	3.5	100	1.0	0.5
0.553	siteC	1.0	distance	3.5	100	1.0	0.5
0.553	siteC	1.0	distance	3.5	100	1.0	0.5
0.554	siteC	4.0	distance	3.5	100	0.025	0.25
0.554	siteC	1.0	distance	16.0	100	1.0	0.25
0.554	siteC	1.0	distance	16.0	100	0.025	0.5
0.554	siteC	1.0	distance	16.0	100	1.0	0.25
0.554	siteA	1.0	distance	16.0	100	1.0	0.25
0.554	siteA	1.0	distance	16.0	100	1.0	0.25
0.554	siteA	1.0	distance	16.0	100	1.0	0.25
0.554	siteC	1.0	distance	16.0	100	0.025	0.5
0.554	siteC	1.0	distance	16.0	100	0.025	0.5
0.554	siteA	1.0	distance	16.0	100	0.025	0.5
0.554	siteA	1.0	distance	16.0	100	0.025	0.5
0.554	siteC	1.0	distance	16.0	100	1.0	0.25
0.554	siteA	1.0	distance	16.0	100	0.025	0.5
0.555	siteC	4.0	distance	8.0	100	0.025	0.5
0.555	siteC	1.0	distance	8.0	100	1.0	0.25
0.555	siteC	1.0	distance	8.0	100	0.025	1.0
0.555	siteC	1.0	distance	8.0	100	1.0	0.25
0.555	siteA	1.0	distance	8.0	100	0.025	1.0
0.555	siteA	1.0	distance	8.0	100	1.0	0.25
0.555	siteA	1.0	distance	8.0	100	0.025	1.0
0.555	siteC	1.0	distance	8.0	100	0.025	1.0
0.555	siteC	1.0	distance	8.0	100	0.025	1.0
0.555	siteA	1.0	distance	8.0	100	1.0	0.25
0.555	siteA	1.0	distance	8.0	100	0.025	1.0
0.555	siteA	1.0	distance	8.0	100	1.0	0.25
0.555	siteC	1.0	distance	8.0	100	1.0	0.25
0.557	siteC	4.0	distance	3.5	100	0.025	0.5
0.558	siteA	1.0	distance	3.5	100	0.025	1.0
0.558	siteC	1.0	distance	3.5	100	0.025	1.0
0.558	siteA	1.0	distance	3.5	100	0.025	1.0
0.558	siteC	1.0	distance	3.5	100	0.025	1.0
0.558	siteA	1.0	distance	3.5	100	0.025	1.0
0.558	siteC	1.0	distance	3.5	100	0.025	1.0
0.561	siteC	1.0	distance	3.5	100	0.05	1.0
0.561	siteA	1.0	distance	3.5	100	0.05	1.0
0.561	siteA	1.0	distance	3.5	100	0.05	1.0
0.561	siteC	1.0	distance	3.5	100	0.05	1.0
0.561	siteA	1.0	distance	3.5	100	0.05	1.0
0.561	siteC	1.0	distance	3.5	100	0.05	1.0
0.561	siteC	4.0	distance	3.5	100	0.025	1.0
0.57	siteC	4.0	distance	16.0	100	0.05	0.25
0.571	siteC	1.0	distance	3.5	100	1.0	0.25

Continued on next page

Table S-4 – continued from previous page

testMAE	points	siteSize	geometry	gridMax	n	sigma	scale
0.571	siteC	4.0	distance	8.0	100	0.05	1.0
0.571	siteA	1.0	distance	3.5	100	1.0	0.25
0.571	siteA	1.0	distance	3.5	100	1.0	0.25
0.571	siteC	1.0	distance	3.5	100	1.0	0.25
0.571	siteA	1.0	distance	3.5	100	1.0	0.25
0.571	siteC	1.0	distance	3.5	100	1.0	0.25
0.572	molC	4.0	inverse	20.0	100	0.025	0.0
0.572	siteC	4.0	distance	16.0	100	0.025	0.25
0.576	siteC	4.0	distance	16.0	100	0.05	0.5
0.577	siteC	1.0	distance	16.0	100	0.05	1.0
0.577	siteC	1.0	distance	16.0	100	0.05	1.0
0.577	siteA	1.0	distance	8.0	100	1.0	1.0
0.577	siteC	1.0	distance	8.0	100	1.0	1.0
0.577	siteA	1.0	distance	16.0	100	0.05	1.0
0.577	siteC	1.0	distance	16.0	100	0.05	1.0
0.577	siteA	1.0	distance	16.0	100	0.05	1.0
0.577	siteA	1.0	distance	16.0	100	0.05	1.0
0.577	siteC	1.0	distance	8.0	100	1.0	1.0
0.577	siteC	1.0	distance	8.0	100	1.0	1.0
0.577	siteA	1.0	distance	8.0	100	1.0	1.0
0.577	siteA	1.0	distance	8.0	100	1.0	1.0
0.578	siteC	4.0	distance	16.0	100	0.025	0.5
0.578	siteC	4.0	distance	8.0	100	0.025	1.0
0.579	siteA	1.0	distance	16.0	100	1.0	1.0
0.579	siteA	1.0	distance	16.0	100	1.0	1.0
0.579	siteC	1.0	distance	16.0	100	1.0	1.0
0.579	siteA	1.0	distance	16.0	100	1.0	1.0
0.579	siteC	1.0	distance	16.0	100	1.0	1.0
0.579	siteC	1.0	distance	16.0	100	1.0	1.0
0.581	siteC	4.0	distance	3.5	100	0.05	0.25
0.585	siteC	1.0	distance	16.0	100	0.025	1.0
0.585	siteC	1.0	distance	16.0	100	0.025	1.0
0.585	siteA	1.0	distance	16.0	100	0.025	1.0
0.585	siteA	1.0	distance	16.0	100	0.025	1.0
0.585	siteC	4.0	distance	3.5	100	0.05	0.5
0.585	siteC	1.0	distance	16.0	100	0.025	1.0
0.585	siteA	1.0	distance	16.0	100	0.025	1.0
0.586	siteA	1.0	distance	3.5	100	1.0	1.0
0.586	siteC	1.0	distance	3.5	100	1.0	1.0
0.586	siteC	1.0	distance	3.5	100	1.0	1.0
0.586	siteC	1.0	distance	3.5	100	1.0	1.0
0.586	siteA	1.0	distance	3.5	100	1.0	1.0
0.586	siteA	1.0	distance	3.5	100	1.0	1.0
0.587	molC	4.0	distance	10.0	500	1.0	0.5
0.59	siteC	4.0	distance	3.5	100	0.05	1.0

Continued on next page

Table S-4 – continued from previous page

testMAE	points	siteSize	geometry	gridMax	n	sigma	scale
0.591	siteC	4.0	inverse	20.0	100	0.05	0.0
0.591	molC	4.0	inverse	20.0	100	0.001	0.0
0.591	molC	4.0	inverse	20.0	100	0.0001	0.0
0.598	siteC	4.0	distance	16.0	100	0.05	1.0
0.6	siteC	4.0	distance	16.0	100	0.025	1.0
0.613	molC	1.0	distance	16.0	100	1.0	1.0
0.614	molC	1.0	distance	8.0	100	1.0	1.0
0.618	molC	1.0	distance	3.5	100	1.0	1.0
0.624	siteC	4.0	inverse	20.0	100	0.1	0.0
0.628	molC	1.0	distance	16.0	100	1.0	0.5
0.629	molC	1.0	distance	8.0	100	1.0	0.5
0.629	molC	1.0	distance	16.0	100	1.0	0.25
0.629	molC	1.0	distance	8.0	100	1.0	0.25
0.63	molC	1.0	distance	3.5	100	1.0	0.5
0.633	siteC	4.0	distance	16.0	100	1.0	0.25
0.633	siteC	4.0	distance	8.0	100	1.0	0.25
0.634	molC	1.0	distance	3.5	100	1.0	0.25
0.644	siteC	4.0	distance	16.0	100	1.0	0.5
0.644	siteC	4.0	distance	8.0	100	1.0	0.5
0.648	siteC	4.0	distance	10.0	500	1.0	0.5
0.666	siteC	4.0	distance	3.5	100	1.0	0.25
0.672	siteC	4.0	distance	8.0	100	1.0	1.0
0.672	siteC	4.0	distance	16.0	100	1.0	1.0
0.696	siteC	4.0	distance	3.5	100	1.0	0.5
0.73	siteC	4.0	distance	10.0	500	2.0	0.5
0.746	siteC	4.0	distance	3.5	100	1.0	1.0

S-5.3 LMBTR k=3 Optimization

Table S-5. Results for the optimization of the parameters of the LMBTR with $k = 3$ descriptor using the linear regression.

testMAE	points	siteSize	geometry	gridMax	n	sigma	scale
0.237	molC	3.0	cosine	2.0	20	0.1	0.0
0.24	molC,siteC	10.0	cosine	2.0	10	0.1	0.0
0.241	molC,siteC	8.0	cosine	2.0	10	0.15	0.0
0.242	molC	4.0	cosine	2.0	20	0.1	0.0
0.242	molC,inter	8.0	cosine	2.0	10	0.15	0.0
0.245	molC,inter	10.0	cosine	2.0	10	0.1	0.0
0.247	molC	5.0	cosine	2.0	20	0.1	0.0
0.252	molC	3.0	cosine	2.0	20	0.15	0.0
0.253	molC,siteC	10.0	cosine	2.0	10	0.15	0.0
0.257	molC,siteC	8.0	cosine	2.0	10	0.2	0.0
0.257	molC	4.0	cosine	2.0	20	0.15	0.0
0.258	molC,siteC,inter	8.0	cosine	2.0	10	0.1	0.0

Continued on next page

Table S-5 – continued from previous page

testMAE	points	siteSize	geometry	gridMax	n	sigma	scale
0.259	molC	6.0	cosine	2.0	20	0.1	0.0
0.261	molC,siteC,inter	2.0	cosine	2.0	10	0.1	0.0
0.262	molC,siteC,inter	6.0	cosine	2.0	10	0.1	0.0
0.263	molC,siteC,inter	4.0	cosine	2.0	10	0.1	0.0
0.263	molC	5.0	cosine	2.0	20	0.15	0.0
0.264	inter	5.0	cosine	2.0	20	0.1	0.0
0.267	inter	3.0	cosine	2.0	20	0.1	0.0
0.268	molC,siteC	1.0	cosine	2.0	10	0.1	0.0
0.269	inter	4.0	cosine	2.0	20	0.1	0.0
0.273	molC,siteC	1.0	cosine	2.0	10	0.05	0.0
0.274	molC	6.0	cosine	2.0	20	0.15	0.0
0.276	molC,siteC,inter	8.0	cosine	2.0	10	0.15	0.0
0.277	molC,siteC	10.0	cosine	2.0	10	0.1	0.0
0.278	molC,siteC	1.0	cosine	2.0	10	0.025	0.0
0.278	inter	3.0	cosine	2.0	20	0.15	0.0
0.278	molC,siteC	2.0	cosine	2.0	10	0.1	0.0
0.278	molC,siteC	8.0	cosine	2.0	10	0.1	0.0
0.279	molC,siteC,inter	2.0	cosine	2.0	10	0.15	0.0
0.279	inter	6.0	cosine	2.0	20	0.1	0.0
0.28	molC,siteC,inter	6.0	cosine	2.0	10	0.15	0.0
0.28	inter	5.0	cosine	2.0	20	0.15	0.0
0.28	molC,inter	8.0	cosine	2.0	10	0.1	0.0
0.281	molC,siteC	6.0	cosine	2.0	10	0.1	0.0
0.282	molC,inter	10.0	cosine	2.0	10	0.1	0.0
0.282	molC,siteC,inter	4.0	cosine	2.0	10	0.15	0.0
0.283	molC	1.0	cosine	2.0	10	0.1	0.0
0.283	inter	4.0	cosine	2.0	20	0.15	0.0
0.283	molC,siteC	4.0	cosine	2.0	10	0.1	0.0
0.285	molC,inter	2.0	cosine	2.0	10	0.1	0.0
0.285	molC,inter	6.0	cosine	2.0	10	0.1	0.0
0.286	molC	1.0	cosine	2.0	10	0.05	0.0
0.286	molC,inter	4.0	cosine	2.0	10	0.1	0.0
0.292	molC	1.0	cosine	2.0	10	0.025	0.0
0.294	molC,siteC	2.0	cosine	2.0	10	0.15	0.0
0.295	molC,siteC	10.0	cosine	2.0	10	0.15	0.0
0.296	molC,siteC	8.0	cosine	2.0	10	0.15	0.0
0.298	molC,inter	8.0	cosine	2.0	10	0.15	0.0
0.298	molC,inter	10.0	cosine	2.0	10	0.15	0.0
0.298	molC,siteC	6.0	cosine	2.0	10	0.15	0.0
0.301	molC	4.0	cosine	2.0	20	0.025	0.0
0.301	molC,siteC	4.0	cosine	2.0	10	0.15	0.0
0.303	molC,inter	2.0	cosine	2.0	10	0.15	0.0
0.303	molC,inter	6.0	cosine	2.0	10	0.15	0.0
0.304	molC,inter	4.0	cosine	2.0	10	0.15	0.0
0.339	molC	4.0	cosine	2.0	20	0.1	0.0

Continued on next page

Table S-5 – continued from previous page

testMAE	points	siteSize	geometry	gridMax	n	sigma	scale
0.341	molC,siteC	1.0	cosine	2.0	10	1.0	0.0
0.349	molC	4.0	cosine	2.0	10	0.005	0.0
0.357	molC	1.0	cosine	2.0	10	1.0	0.0
0.405	siteC	1.0	cosine	2.0	10	0.1	0.0
0.42	siteC	1.0	cosine	2.0	10	0.05	0.0
0.437	siteC	1.0	cosine	2.0	10	0.025	0.0
0.518	siteC	1.0	cosine	2.0	10	1.0	0.0

S-5.4 Optuna Hyperparameter Optimization

Tables S-6 and S-7 presents the achieved MAE values for the validation set during the Optuna hyperparameter optimization using both CM and LMBTR descriptors, respectively. The opimized parameters are trees maximum depth (`max_depth`), ratio of used features for fitting trees (`max_features`), minimum samples for a leaf node (`min_samples_leaf`, minimum samples required for a split (`min_samples_split`), number of trees (`n_estimators`).

S-5.4.1 RFR + CM Optimization

Table S-6. Results for the RFR hyperparameter optimization for the optimized CM.

MAE	max_depth	max_features	min_samples_leaf	min_samples_split	n_estimators
0.048	78.0	0.64	1.0	8.0	100.0
0.048	73.0	0.63	1.0	8.0	102.0
0.049	76.0	0.67	1.0	11.0	102.0
0.049	49.0	0.88	2.0	9.0	135.0
0.049	75.0	0.7	1.0	11.0	102.0
0.049	80.0	0.76	1.0	11.0	117.0
0.049	79.0	0.48	1.0	10.0	101.0
0.049	80.0	0.8	1.0	12.0	108.0
0.049	49.0	0.77	2.0	10.0	162.0
0.05	50.0	0.79	2.0	11.0	160.0
0.05	75.0	0.63	3.0	9.0	67.0
0.05	65.0	0.51	3.0	9.0	93.0
0.05	46.0	0.77	2.0	13.0	159.0
0.05	39.0	0.98	1.0	14.0	141.0
0.05	68.0	0.74	4.0	8.0	79.0
0.05	64.0	0.47	1.0	13.0	150.0
0.05	33.0	0.99	1.0	14.0	171.0
0.051	58.0	0.58	4.0	8.0	117.0
0.051	34.0	0.96	1.0	14.0	139.0
0.051	62.0	0.41	3.0	10.0	117.0
0.051	44.0	0.63	3.0	13.0	144.0
0.051	31.0	0.82	3.0	14.0	170.0
0.053	54.0	0.82	5.0	12.0	157.0

Continued on next page

Table S-6 – continued from previous page

MAE	max_depth	max_features	min_samples_leaf	min_samples_split	n_estimators
0.053	32.0	0.68	6.0	8.0	145.0
0.053	68.0	0.44	5.0	12.0	97.0
0.053	63.0	0.69	4.0	17.0	151.0
0.054	44.0	0.92	6.0	15.0	171.0
0.055	77.0	0.46	4.0	19.0	196.0
0.055	50.0	0.79	7.0	10.0	182.0
0.055	73.0	0.66	7.0	11.0	70.0
0.055	73.0	0.13	1.0	8.0	96.0
0.055	51.0	0.38	3.0	19.0	132.0
0.055	55.0	0.59	7.0	16.0	81.0
0.056	25.0	0.84	6.0	19.0	145.0
0.056	61.0	0.89	9.0	9.0	199.0
0.056	61.0	0.48	7.0	13.0	167.0
0.057	48.0	0.56	8.0	13.0	51.0
0.057	59.0	0.58	8.0	18.0	181.0
0.058	67.0	0.11	1.0	12.0	81.0
0.058	80.0	0.24	4.0	8.0	93.0
0.059	53.0	0.7	10.0	17.0	150.0
0.06	64.0	0.98	12.0	19.0	177.0
0.061	77.0	0.58	11.0	16.0	121.0
0.061	37.0	0.32	9.0	13.0	133.0
0.062	54.0	1.0	14.0	15.0	155.0
0.063	45.0	0.85	14.0	11.0	56.0
0.064	44.0	0.32	10.0	19.0	65.0
0.066	22.0	0.97	15.0	12.0	192.0
0.067	40.0	0.38	15.0	16.0	127.0
0.067	37.0	0.3	12.0	10.0	140.0
0.069	71.0	0.52	20.0	8.0	50.0
0.07	23.0	0.88	19.0	15.0	106.0
0.07	23.0	0.73	20.0	16.0	189.0
0.071	74.0	0.36	18.0	11.0	196.0
0.073	21.0	0.41	15.0	8.0	127.0
0.074	38.0	0.22	6.0	20.0	157.0
0.082	54.0	0.11	3.0	10.0	106.0
0.096	71.0	0.22	19.0	14.0	90.0
0.159	12.0	1.0	9.0	13.0	116.0
0.164	12.0	0.61	5.0	20.0	85.0

S-5.4.2 RFR + LMBTR Optimization

Table S-7. Results for the RFR hyperparameter optimization for the optimized LMBTR.

MAE	max_depth	max_features	min_samples_leaf	min_samples_split	n_estimators
0.048	78.0	0.64	1.0	8.0	100.0
0.048	73.0	0.63	1.0	8.0	102.0

Continued on next page

Table S-7 – continued from previous page

MAE	max_depth	max_features	min_samples_leaf	min_samples_split	n_estimators
0.049	76.0	0.67	1.0	11.0	102.0
0.049	49.0	0.88	2.0	9.0	135.0
0.049	75.0	0.7	1.0	11.0	102.0
0.049	80.0	0.76	1.0	11.0	117.0
0.049	79.0	0.48	1.0	10.0	101.0
0.049	80.0	0.8	1.0	12.0	108.0
0.049	49.0	0.77	2.0	10.0	162.0
0.05	50.0	0.79	2.0	11.0	160.0
0.05	75.0	0.63	3.0	9.0	67.0
0.05	65.0	0.51	3.0	9.0	93.0
0.05	46.0	0.77	2.0	13.0	159.0
0.05	39.0	0.98	1.0	14.0	141.0
0.05	68.0	0.74	4.0	8.0	79.0
0.05	64.0	0.47	1.0	13.0	150.0
0.05	33.0	0.99	1.0	14.0	171.0
0.051	58.0	0.58	4.0	8.0	117.0
0.051	34.0	0.96	1.0	14.0	139.0
0.051	62.0	0.41	3.0	10.0	117.0
0.051	44.0	0.63	3.0	13.0	144.0
0.051	31.0	0.82	3.0	14.0	170.0
0.053	54.0	0.82	5.0	12.0	157.0
0.053	32.0	0.68	6.0	8.0	145.0
0.053	68.0	0.44	5.0	12.0	97.0
0.053	63.0	0.69	4.0	17.0	151.0
0.054	44.0	0.92	6.0	15.0	171.0
0.055	77.0	0.46	4.0	19.0	196.0
0.055	50.0	0.79	7.0	10.0	182.0
0.055	73.0	0.66	7.0	11.0	70.0
0.055	73.0	0.13	1.0	8.0	96.0
0.055	51.0	0.38	3.0	19.0	132.0
0.055	55.0	0.59	7.0	16.0	81.0
0.056	25.0	0.84	6.0	19.0	145.0
0.056	61.0	0.89	9.0	9.0	199.0
0.056	61.0	0.48	7.0	13.0	167.0
0.057	48.0	0.56	8.0	13.0	51.0
0.057	59.0	0.58	8.0	18.0	181.0
0.058	67.0	0.11	1.0	12.0	81.0
0.058	80.0	0.24	4.0	8.0	93.0
0.059	53.0	0.7	10.0	17.0	150.0
0.06	64.0	0.98	12.0	19.0	177.0
0.061	77.0	0.58	11.0	16.0	121.0
0.061	37.0	0.32	9.0	13.0	133.0
0.062	54.0	1.0	14.0	15.0	155.0
0.063	45.0	0.85	14.0	11.0	56.0
0.064	44.0	0.32	10.0	19.0	65.0

Continued on next page

Table S-7 – continued from previous page

MAE	max_depth	max_features	min_samples_leaf	min_samples_split	n_estimators
0.066	22.0	0.97	15.0	12.0	192.0
0.067	40.0	0.38	15.0	16.0	127.0
0.067	37.0	0.3	12.0	10.0	140.0
0.069	71.0	0.52	20.0	8.0	50.0
0.07	23.0	0.88	19.0	15.0	106.0
0.07	23.0	0.73	20.0	16.0	189.0
0.071	74.0	0.36	18.0	11.0	196.0
0.073	21.0	0.41	15.0	8.0	127.0
0.074	38.0	0.22	6.0	20.0	157.0
0.082	54.0	0.11	3.0	10.0	106.0
0.096	71.0	0.22	19.0	14.0	90.0
0.159	12.0	1.0	9.0	13.0	116.0
0.164	12.0	0.61	5.0	20.0	85.0

References

1 Blum, V.; Gehrke, R.; Hanke, F.; Havu, P.; Havu, V.; Ren, X.; Reuter, K.; Scheffler, M. *Ab initio* Molecular Simulations With Numeric Atom-centered Orbitals. *Comput. Phys. Commun.* **2009**, *180*, 2175–2196, DOI: 10.1016/j.cpc.2009.06.022.

2 Andriani, K. F.; Mucelini, J.; Da Silva, J. L. F. Methane dehydrogenation on 3d 13-atom transition-metal clusters: A density functional theory investigation combined with Spearman rank correlation analysis. *Fuel* **2020**, *275*, 117790, DOI: 10.1016/j.fuel.2020.117790.

3 Felício-Sousa, P.; Andriani, K. F.; Da Silva, J. L. F. Ab initio investigation of the role of the d-states occupation on the adsorption properties of H_2, CO, CH_4 and CH_3OH on the Fe_{13}, Co_{13}, Ni_{13} and Cu_{13} clusters. *Phys. Chem. Chem. Phys.* **2021**, *23*, 8739–8751, DOI: 10.1039/d0cp06091g.

4 Andriani, K. F.; Felício-Sousa, P.; Morais, F. O.; Da Silva, J. L. F. Role of quantum-size effects in the dehydrogenation of CH_4 on 3*d* TM_n clusters: DFT calculations combined with data mining. *Catalysis Science & Technology* **2022**, *12*, 916–926, DOI: 10.1039/d1cy01785c.

5 Collacique, M. N.; Ocampo-Restrepo, V. K.; Da Silva, J. L. F. Ab initio investigation of the role of the *d*-states on the adsorption and activation properties of CO_2 on 3*d*, 4*d*, and 5*d* transition-metal clusters. *J. Chem. Phys.* **2022**, *156*, 124106, DOI: 10.1063/5.0085364.

6 De Sousa, R. A.; Ocampo-Restrepo, V. K.; Verga, L. G.; Da Silva, J. L. F. Ab initio study of the adsorption properties of CO_2 reduction intermediates: The effect of Ni_5Ga_3 alloy and the Ni_5Ga_3/ZrO_2 interface. *J. Chem. Phys.* **2022**, *156*, 214106, DOI: 10.1063/5.0091145.

7 Gomes, I. L.; Da Silva, J. L. F. Investigação Teórica da Adsorção de Moléculas sobre Nanocluster de Óxido de Zircônia. Ph.D. thesis, 2022.

8 Ocampo-Restrepo, V. K.; Verga, L. G.; Da Silva, J. L. F. Ab initio study for late steps of CO_2 and CO electroreduction: from $CHCO^*$ toward C_2 products on Cu and CuZn nanoclusters. *Phys. Chem. Chem. Phys.* **2023**, *25*, 32931–32938, DOI: 10.1039/d3cp03315e.

9 Felício-Sousa, P. Investigação Ab initio da ativação de metano em clusters metálicos e óxidos: integração com mineração de dados. Ph.D. thesis, São Carlos Institute of Chemistry, 2024.

10 Bro, R.; Smilde, A. K. Principal component analysis. *Anal. Methods* **2014**, *6*, 2812–2831, DOI: 10.1039/c3ay41907j.

11 Himanen, L.; Jäger, M. O.; Morooka, E. V.; Federici Canova, F.; Ranawat, Y. S.; Gao, D. Z.; Rinke, P.; Foster, A. S. DScribe: Library of descriptors for machine learning in materials science. *Comput. Phys. Commun.* **2020**, *247*, 106949, DOI: 10.1016/j.cpc.2019.106949.
